# Landscape genomics reveals regions associated with adaptive phenotypic and genetic variation in Ethiopian indigenous chickens

**DOI:** 10.1186/s12864-024-10193-6

**Published:** 2024-03-18

**Authors:** Fasil Getachew Kebede, Martijn F.L. Derks, Tadelle Dessie, Olivier Hanotte, Carolina Pita Barros, Richard P.M.A. Crooijmans, Hans Komen, John W.M. Bastiaansen

**Affiliations:** 1 Animal Breeding and Genomics, Wageningen University & Research, Droevendaalsesteeg 1, Wageningen, PB-6708 The Netherlands; 2 International Livestock Research Institute, P.O. Box 5689, Addis Ababa, Ethiopia; 3 School of Life Sciences, The University of Nottingham, Nottingham, NG7 2RD UK

**Keywords:** Local adaptation, Poultry production, Signatures of selection, Redundancy analysis, Environmental predictors, Quantitative traits, Genetic improvement

## Abstract

Climate change is a threat to sustainable livestock production and livelihoods in the tropics. It has adverse impacts on feed and water availability, disease prevalence, production, environmental temperature, and biodiversity. Unravelling the drivers of local adaptation and understanding the underlying genetic variation in random mating indigenous livestock populations informs the design of genetic improvement programmes that aim to increase productivity and resilience. In the present study, we combined environmental, genomic, and phenotypic information of Ethiopian indigenous chickens to investigate their environmental adaptability. Through a hybrid sampling strategy, we captured wide biological and ecological variabilities across the country. Our environmental dataset comprised mean values of 34 climatic, vegetation and soil variables collected over a thirty-year period for 260 geolocations. Our biological dataset included whole genome sequences and quantitative measurements (on eight traits) from 513 individuals, representing 26 chicken populations spread along 4 elevational gradients (6–7 populations per gradient). We performed signatures of selection analyses ($$ {F}_{ST}$$ and XP-EHH) to detect footprints of natural selection, and redundancy analyses (RDA) to determine genotype-environment and genotype-phenotype-associations. RDA identified 1909 outlier SNPs linked with six environmental predictors, which have the highest contributions as ecological drivers of adaptive phenotypic variation. The same method detected 2430 outlier SNPs that are associated with five traits. A large overlap has been observed between signatures of selection identified by$$ { F}_{ST }$$and XP-EHH showing that both methods target similar selective sweep regions. Average genetic differences measured by $$ {F}_{ST}$$ are low between gradients, but XP-EHH signals are the strongest between agroecologies. Genes in the calcium signalling pathway, those associated with the hypoxia-inducible factor (HIF) transcription factors, and sports performance (*GALNTL6)* are under selection in high-altitude populations. Our study underscores the relevance of landscape genomics as a powerful interdisciplinary approach to dissect adaptive phenotypic and genetic variation in random mating indigenous livestock populations.

**Supplementary Information:**

The online version contains supplementary material available at 10.1186/s12864-024-10193-6.

## Background

The genetics of local adaptation and climate resilience in livestock has become more relevant in the face of climate change [1–4]. Local adaptation refers to the response of individuals to differential selective pressure leading to higher genetic fitness in their environment than individuals from elsewhere [5, 6]. Resilient animals have the capacity to be minimally affected by environmental disturbances (e.g., temperature stress, disease pressure, introduction to a new habitat) if they occur, or can return rapidly to the state pertained before exposure to the disturbance [7, 8]. Animals that combine high production potential with resilience to external stressors in a wide variety of environmental conditions are regarded as ‘robust’ [9].

Phenotypic differentiation represents the fraction of phenotypic variance between populations over the total phenotypic variance and helps understand evolutionary processes shaping populations [10–12]. Environmental differences acting as a natural selective force can result in exceptionally strong genetic differentiation in genomic regions containing loci subjected to selection [13]. Phenotypic and genetic differentiation along environmental gradients, or across contrasting habitat types, can be indicative of local adaptation [6, 14, 15]. For instance, alleles providing adaptation to high elevation are found in high frequency in populations at high elevation but in low frequency in populations at low elevation in humans [16, 17], in chickens [16, 18, 19], in pigs [20, 21], and small ruminants [22].

Understanding the genetic basis of phenotypic variation and local adaptation in livestock in response to environmental variation helps to enhance productivity and mitigate climate change [2, 23–25]. Randomly mating indigenous livestock populations are raised in stressful environmental conditions for many generations and harbour genomic regions conferring local adaptation that need to be exploited [26, 27]. Once identified, beneficial alleles/variants in indigenous chickens can be introduced into commercial chickens through breeding programmes [28, 29] or genome editing [30–32] to develop animals with desirable phenotypic attributes.

The effects of environmental selective pressures as drivers of local adaptation and specially their influences on phenotypic and genetic differentiation in Ethiopian chicken populations have not been investigated enough to shape our understanding of environmental adaptation. Ethiopian indigenous chickens also called local, village, scavenging, backyard, or family chickens are widely adapted, random mating, nondescript domesticated chicken populations. They are managed in extensive (low-input) systems in their natural environment, without selective breeding programmes in place [33]. Ethiopia has one of the earliest evidences for chicken domestication and dispersal in Africa [34]. Ethiopian chickens are distributed in all agroecologies [33] and show substantial phenotypic and genetic diversity [35–39]. Large genetic diversity of present-day Ethiopian chicken populations might be attributed to their multiple waves of introduction into the country [40, 41] and the presence of highly diverse environment (e.g., climate, vegetation, elevation) [42]. As such, the country can be considered an ideal place for studying adaptive phenotypic and genetic variation in chickens.

Certain phenotypes in Ethiopian indigenous chickens (e.g., comb shape, parasitic resistance) are related with local adaptation [26]. Genomic regions conferring adaptation to environmental challenges (e.g., elevation, temperature, water scarcity, and feed availability) have been identified among African indigenous chickens [43–45]. Important insights on local adaptation of Ethiopian indigenous chickens were obtained in previous studies: the association between environmental predictors and phenotypic differentiation in quantitative traits was reported as an evidence for adaptative variation [46]. Another important study claimed that environmental conditions may have driven genomic variation in indigenous chicken populations [47].

At an interface between ecology and population genetics, landscape genomics provides an analytical framework useful to investigate the underlying evolutionary processes behind phenotypic and genetic differentiation of random mating populations raised in heterogenous environments. Landscape genomics seeks to understand the influences of geographic and environmental features on selectively neutral and adaptive loci, and underlying micro-evolutionary processes such as gene flow, selection, and genetic drift [48, 49]. Landscape genomic approaches were followed in studies of adaptive genetic variation in different farm animal species animals including chickens, sheep, goats, swine, and cattle [45, 50–57].

Popular tools being used in landscape genomic studies include species distribution modelling, signatures of selection analyses, and genotype-environment analyses. Multivariate methods that simultaneously account for multiple drivers of phenotypic and environmental divergence, are recently being applied in landscape genomic studies to identify quantitative trait loci (QTL) associated with environment predictors [58–61] and with phenotypic variables [60, 62–65].

Species distribution models (SDMs), also known as environmental (ecological) niche models (ENMs) or habitat distribution models [66], use computer algorithms to analyse environmental data and to predict the distribution of a species across geographic space and time. SDMs are a popular tool in quantitative ecology because of their low data requirement, availability of many software packages and guidelines, and their higher predictive abilities [67, 68]. The central concept in SDMs is the niche theory [69, 70], which delineates the environment into fundamental and realized niches. In recent years, the conceptual framework for SDMs has been extended by livestock scientists and used to identify environmental predictors associated with habitat suitability and local adaptation [45, 46, 56, 71, 72].

Signatures of selection analysis are useful to identify regions of the genome that have differentiated between populations, possibly in response to selective pressure [73, 74]. Positive selection leaves conspicuous footprints or selective sweeps on the genome that can be detected using several approaches ranging from summary statistics such as Tajima’s D, to maximum likelihood and machine learning [75]. Cross-population Extended Haplotype Homozygosity (XP-EHH) detects differential selection between two populations [76]. Pairwise comparison of fixation index ($$ {F}_{ST}$$) reveals differentiation of populations in different environments due to differences in evolutionary history [77]. $$ {F}_{ST }$$and XP-EHH approaches are complementary to each other and lead to a more comprehensive understanding of signatures of selection. $$ {F}_{ST }$$is more suited for detection of positive selection in the distant past [78] while XP-EHH is more useful for detection of entirely or approximately fixed loci [76].

Another statistical method that is being used in landscape genomics is Redundancy Analysis (RDA). RDA is useful to investigate association between genomic and environmental variability. RDA combines regression and principal component analysis (PCA) and it is an extremely powerful tool for ecologists to model multivariate response data [79, 80]. RDA determines how groups of loci covary in response to the multivariate environment, and can better detect processes that result in weak, multilocus molecular signatures relative to univariate tests [81]. It accounts for population structures, demographic histories, and polygenic interactions [59, 82]. Multivariate methods like RDA, that simultaneously account for multiple drivers of phenotypic and environmental divergence are being used to identify quantitative trait loci (QTL) associated with environment predictors [58–61]. Multivariate ordination methods such as RDA have outperformed mixed-model-based methods and machine learning-based methods (e.g., Random Forest) in detecting loci associated with environmental variation [59, 82]. Despite its ability to investigate genotype-phenotype associations RDA is mostly neglected in GWAS studies in livestock, while it became a standard in genotype-environment association studies in wildlife [62, 83]. In the present study we follow a landscape genomic approach to dissect adaptive genetic and phenotypic variation in Ethiopian indigenous chickens. We use SDMs to identify the most relevant environmental predictors driving local adaptation and produce habitat suitability maps. We perform signatures of selection analyses ($$ {F}_{ST}$$ and XP-EHH) to detect genetic differentiation between populations and selective sweeps. RDA is applied to identify outlier SNPs associated with environmental and phenotypic variation. Together, we explain variations in the genome by using key environmental drivers and identify candidate genes and genomic regions linked with environmental adaptation in Ethiopian indigenous chickens.

## Materials and methods

### Sampling strategy

Sampling design is a fundamental aspect of landscape genomic studies. As such, we implemented a robust sampling strategy, considering environmental gradation (e.g., elevational clines) and geographic (latitudinal and longitudinal) variation in the country [46] to avoid biases in discovery of genomic regions under selection. A hybrid strategy combines maximization of geographic distance (based on coordinates) and climatic distance between chosen sites. The landscape is divided into distinct environmental regions before choosing sites within each region that maximizes spatial distance [84]. A hybrid sampling strategy ensures environmental and geographic representativeness of sampling sites and increases statistical power by reducing false discovery rates of statistically significant loci in signatures of selections analysis and genome wide association studies [84–86]. The strategy also prevents the sampling of neighbouring sites with similar conditions and avoids the superposition between adaptive and neutral genetic variation [87].

The spatial distribution of our samples considered environmental (e.g., geography, climate) and biotic processes (e.g., domestication, routes of introduction) influencing the chicken populations. A total of 513 chickens were sampled from four environmental gradients (*gradient-I, -II, -III, and -IV*) with a minimum distance between gradients of 500 km. Environmental or ecological gradients refer to gradual changes in abiotic environmental factors (such as elevation, temperature, soil, vegetation, and precipitation) with consequences on the species’ distribution and local adaptation [88]. *Gradient-I* stretches from the Rift valley lowlands of northeastern Ethiopia along the territories of Afar region to the highlands of Wollo province within Amhara region. *Gradient-II*, starts from the Rift valley lowlands in central Ethiopia, crosses the highlands of Hararghe, including Mount Gara Muleta, and stretches to eastern Ethiopia within Oromia region. *Gradient-III* stretches from the highlands of northwestern Ethiopia and goes down to the lowlands along the Ethiopian-Sudanese border within Benishangul-Gumuz region. *Gradient-IV* extends from the highlands of western Ethiopia in Oromia region to the lowlands along the Ethiopian-Kenyan border in Southern region. Areas around the national borders of Ethiopia have low elevation, which gradually culminates to highland plateau in the center of the country creating a striking contrast in agroecology.

**Fig. 1:**
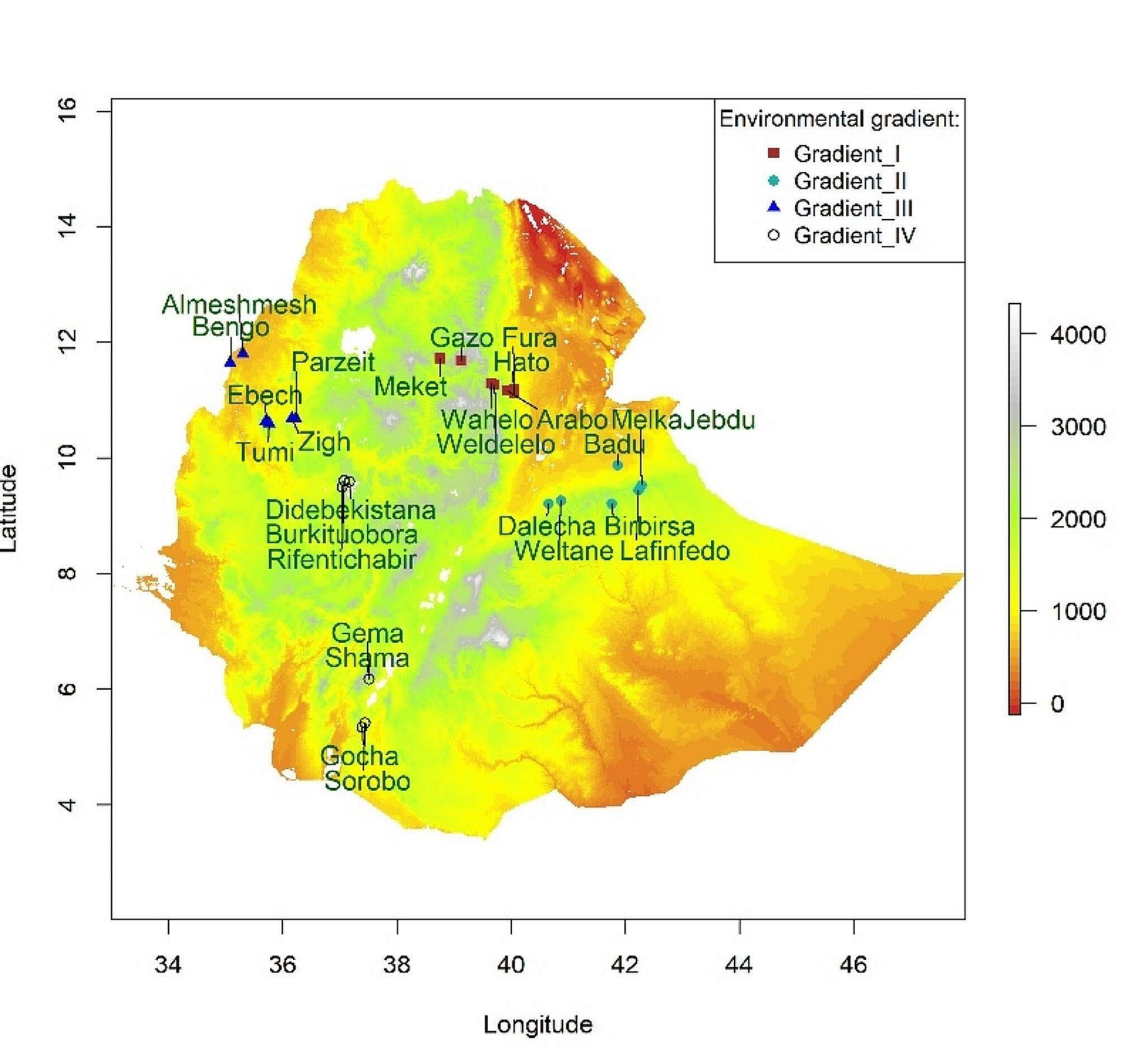
Topographic map of Ethiopia depicting the 26 Ethiopian indigenous chicken sample populations and their environmental gradients. Range of numbers with different colours in the legend indicate elevation (m.a.s.l.)

Each gradient comprised three environmental clusters or agroecologies, primarily delineated based on elevation in meters above sea level (m.a.s.l). These are *lowland* (400–1800 m.a.s.l); *midaltitude/midland* (1800–2400 m.a.s.l.); and *highland* (2400–3500 m.a.s.l.) according to the conventional agroecological classification in Ethiopia [89, 90]. Clusters within a gradient were distant by at least 100 km and farmers keeping target chicken populations within a cluster visited separate livestock markets. Each cluster along the spatial gradient constituted of 2–3 populations. In the context of the present study, a population of Ethiopian indigenous chickens refers to individuals that are kept within a specific geographic area (at *village* level) and which are assumed to be similarly influenced by environmental (ecological) and socio-economic factors. A village (*kebele*) is the smallest administrative unit in Ethiopia. The metadata of 513 individual samples representing 26 chicken populations is presented in Supplementary Table 1 The topographic map of Ethiopia showing the Ethiopian indigenous sample populations and their environmental gradients is presented in Fig. 1.

**Fig. 2:**
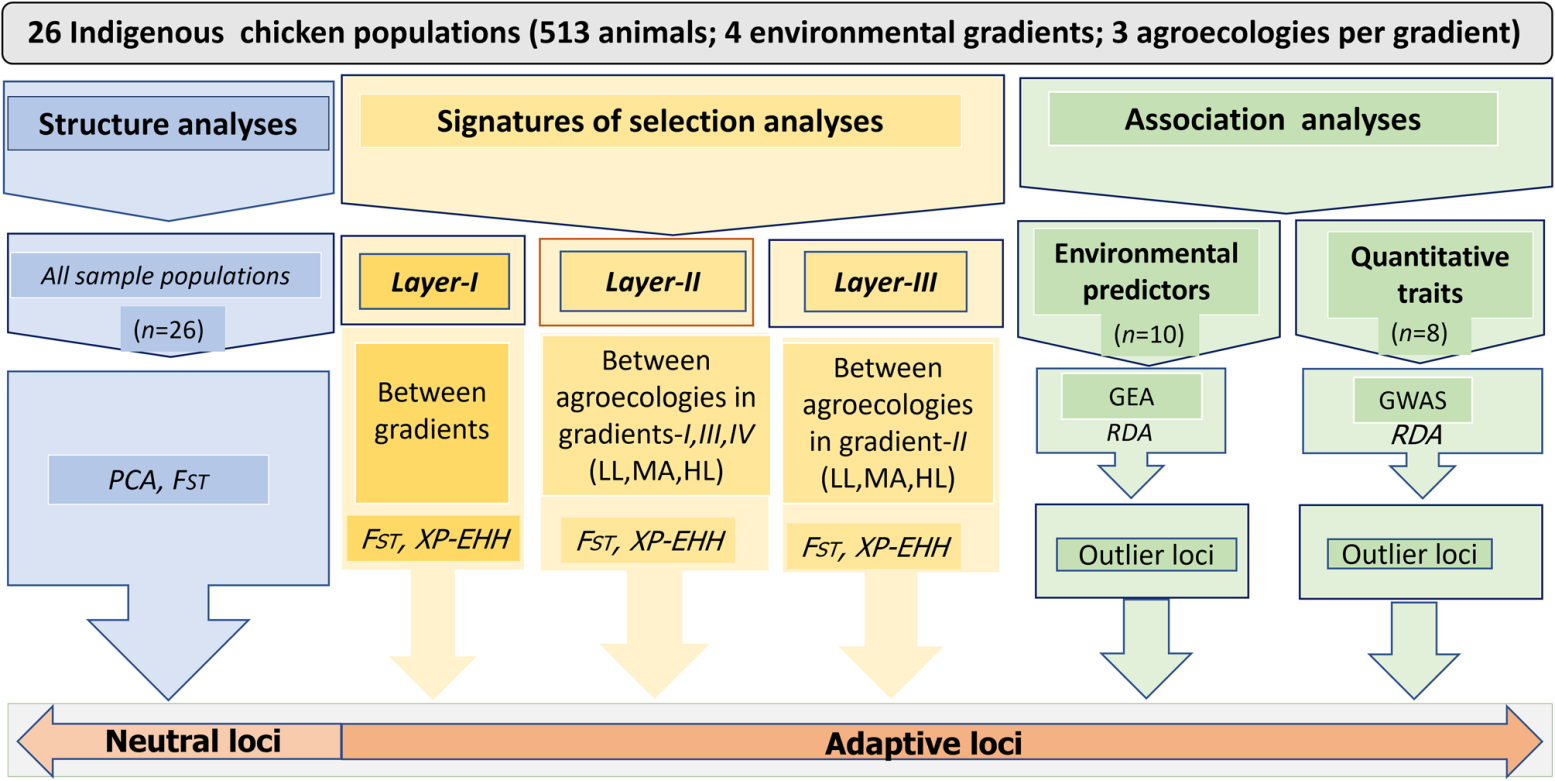
Sampling and analytical framework in landscape genomics study to detect adaptive phenotypic and genetic variation in Ethiopian indigenous chicken populations LL = lowland; MA = midaltitude; and HL = highland. Adaptive loci are the result of natural selection and contribute to fitness while neutral loci are due to other evolutionary process (e.g., gene flow, genetic drift, demographic history)

The chicken populations from different geographies of Ethiopia may be the result of different evolutionary histories. We controlled for the potential confounding effects between demographic processes (e.g., domestication history, migration) and adaptive variation in our analysis by performing signatures of selection analyses at three different analytical layers (*layer-I*, *layer-II, and layer-III*). Figure 2 shows the sampling and analytical framework used in the present study.

### Environmental data

For every population, a single geographic coordinate was taken at the center of the village during sampling of chickens. Coordinates from nine additional grids (1.44km^2^), covering a total of 12.96 km^2^, were then drawn around a recorded location and extracted using Google Earth Pro v 7.3.2 to ensure high representation of environmental variability affecting the population. The total number of ‘presence’ or ‘occurrence’ points used in SDMs for the 26 sample populations comprised 260 coordinates. Out of 34 environmental predictors, 9 predictors identified through SDMs for their association with habitat suitability and adaptive evolution of chickens in Ethiopia [46] were included in the present study for genotype-environment association analysis with RDA. The 9 predictors are isothermality, temperature seasonality, mean temperature of the coldest quarter, precipitation of the warmest quarter, precipitation of the coldest quarter, solar radiation of the month of May, water vapour pressure of the month of May, water vapour pressure of the month of August, and soil clay content (Supplementary Table 2). Values for bioclimatic variables (temperature, precipitation, soil radiation, and water vapour pressure) in different seasons were obtained from WorldClim database (http://www.worldclim.org/; version 2) at a spatial resolution of 30 s (~1 km^2^) [91] based on mean values of 30 years (1970–2000). Additionally, considering the importance of elevation in the conventional definition of agroecologies in Ethiopia [33], its link with certain adaptive traits in chickens [92], and our sampling design that takes into account elevational clines, we incorporated elevation as a tenth environmental predictor. All the ten predictors were used to produce habitat suitability maps for the 26 sample chicken populations with MaxEnt computer algorithm (version 3.4.1) [93]. Configuration of model parameters for MaxEnt was set based on a previous study [46].

#### Quantitative trait data

Collection of phenotypic data was performed on adult chickens (about 20 chickens sampled from each of the 26 villages). These chickens were selected randomly by walking along a defined path (transect) across an adminstative village and sampling one chicken from each farming household until a total of 15 hens and 5 cocks (roosters) were measured. The age of the chickens was estimated by interviewing owners to confirm that females were in their second clutch (7 to 8 months-of-age) and males were above 12 months-of-age. The researchers also visually appraised roosters for the presence of well-developed spurs. To minimize the risk of inbreeding, one chicken was sampled per household. Under rare circumstances (*n* = 9), two chickens were sampled per household when farmers expressed that their animals have no family relationship, for instance when they were obtained from different sources (e.g., one is hatched at home while the other was bought from the market). 19 quantitative traits were initially measured on each of the 513 adult chickens. Out of these 19 quantitative traits, we used the five traits identified by [46] for their putative roles in local adaptation and usefulness in phenotypic classification of Ethiopian chicken populations (Supplementary Table 3). These are live body weight, beak length, comb width, wattle width, and earlobe width. Live body weight (total mass of an individual in grams before slaughter) was taken using a digital scale in the morning when the animal was fasting i.e., before it was released to scavenge in the backyard. The phenotypic measurements for the other traits were read from pictures of individual chickens and analysed using ImageJ software (version 1.52a) [94]. To reduce systematic error, the same operator measured all chickens, which were held in the same position by a technician. A steel ruler was placed in every picture as a distance reference.

### Blood sampling

Whole blood samples were taken from the wing vein of individual chickens in line with standard procedures [95]. A volume of 50–250 µl of whole blood with anticoagulant (K2EDTA) per sample was put into a cryo-tube filled with 1.5 ml absolute ethanol (100%). Samples were preserved at -20^0^C until DNA extraction and processing.

### Whole genome sequence and data processing

WGS data was generated on Illumina HiSeq2000 platform in paired-end mode with a read length of 150 bp. Reads were quality trimmed using Trimmomatic (Version 0.39) (*ILLUMINACLIP:TruSeq3-PE.fa:2:30:10 LEADING:3 TRAILING:3 SLIDINGWINDOW:4:15 MINLEN:36*) [96]. The average depth of coverage was 8.63 (range: 5.47–14.12) with an average mapping rate of 99.2% (97.05–99.6) and a mapping quality of 33.6 (28.77–34.45) to the GRCg6a reference assembly (Ensemble Gallus_gallus.GRCg6a.dna.toplevel.fa). Genomic analysis was performed on autosomes and non-autosomes.

Freebayes variant calling was run on processed bam files (alignment data) to generate VCF file with the following setting: min-base-quality 10 --min-alternate-fraction 0.2 --haplotype-length 0 --ploidy 2 --min-alternate-count 2 [97]. The ‘min-base-quality 10’ specifies the minimum base quality required for a base to be considered during variant calling. The ‘min-alternate-fraction 0.2’ sets the minimum fraction of reads to 20% supporting the alternate allele for a variant to be called. The ‘haplotype-length 0’ disables the haplotype extension feature, ensuring that all reads are considered independently during variant calling. The ’ploidy 2’ defines the ploidy of the organism being analysed, with a value of 2 indicating diploid. The ‘min-alternate-count 2’ establishes a minimum number of 2 observations supporting an alternate allele required for a variant to be called.

Post processing was performed in BCFtools [98] using vcffilter module of with the setting‘-f ‘QUAL > 20’’. SNPs with low phred quality score (< 20), low call rate (< 0.7), and those within 3 bp of an insertion-deletion (indel) were discarded. Filtering of genotypes was performed [99] prior to downstream analyses to improve data quality. Genotypes were filtered for SNPs not in Hardy-Weinberg equilibrium (*p* < 5 × 10^− 6^), with minor allele frequency (MAF) < 5%. After applying stringent quality filtration on genomes of 513 individuals, we used a clean dataset of 25 M (autosomal and non-autosomal SNPs) from 466 individuals for all downstream analyses (see **Materials and Methods**). Information on genome coverage, mapping rate and quality of samples is presented in Supplementary Table 4.

### Population structure analysis

PCA was performed using the Eigenstrat method, with the smartpca function from Eigensoft v 6.1.4 software [100, 101] to understand the structure of the 26 populations. The VCF files containing the found variants were converted to the eigenstrat format with a python script (https://github.com/CarolinaPB/Bioinfo_scripts/blob/main/vcf2eigenstrat.py).

#### Signatures of selection analysis (SSA)

The search for signals of positive selection ($$ {F}_{ST}$$ or XP-EHH) was carried out on SNP data (*n* = 25 M) that were obtained from WGS after stringent quality filtering. Haplotypes were phased using FastPhase software [102] prior to signatures of selection analyses. To identify candidate loci and genomic regions linked with local adaptation, we performed signatures of selection analyses ($$ {F}_{ST }$$and XP-EHH) at three different analytical layers (Fig. 2**)**.

In *layer-I*, we classified the indigenous chicken populations into four gradients (without regard to their agroecologies) and analysed them to detect genetic differentiation between them. The populations across *gradients (-I, -III, and -IV)* were then categorized by agroecology and analysed (lowland, midaltitude, and highland) in *layer-II.* Chickens have a complex history of introduction and dispersal in Africa at large and in Ethiopia in particular through multiple maritime and/or terrestrial routes [40]. Considering the geographic closeness of *gradient-II* to the Arabian Peninsula, we analysed populations from this gradient (*layer-III)* separately by agroecology (lowland, midaltitude, and highland) to account for possible differences in evolutionary processes from the other three gradients.

Fixation test ($$ {F}_{ST}$$) analysis was conducted using VCFtools v0.1.16, [103], to identify regions of increased genomic differentiation between the classifications defined in the analytical layers. $$ {F}_{ST}$$ value of 0 indicates no differentiation between populations while a value of 1 indicates complete differentiation. Previous works in diverse species including chicken suggest that sliding-window analyses between 20 kb (with 10 kb overlap) and 400 kb (with 200 kb overlap) have considerable power to detect changes in allele frequencies and genomic regions with significant divergence between populations in signatures of selection analysis [104, 105].

We calculated the average $$ { F}_{ST }$$values with overlapping windows of 50 kb (25 kb overlapping). We calculated the average XP-EHH values for the classifications defined in each of the analytical layers. Analysis of genomic regions with signs of recent positive selection with XP-EHH was based on the concept of *extended haplotype homozygosity* (EHH) [76, 106] and was performed on phased haplotypes using R package *rehh* (Version 3.2.2) [107]. First, the data2haplohh function was used to convert the VCF files to a suitable format to be used to compute XP-EHH. Then, XP-EHH was calculated with the ies2xpehh function from the same package.

The same size of overlapping bins (50 kb) was used for XP-EHH analysis to allow comparison with $$ {F}_{ST }.$$ First, the average ($$ {F}_{ST}$$ or XP-EHH) values for all bins in each pairwise comparison in an analytical layer were sorted on their significance. Empirical *p*-values were calculated for both $$ {F}_{ST}$$ and XP-EHH by ranking the windows based on each metric and dividing the rank by the total number of windows. The same approach was used by a previous study on Ethiopian chickens [45]. Only the 1% most significant windows (*p* < 0.01 $$ {F}_{ST }$$ or XP-EHH) were retained as significant. Significant windows which were commonly identified by the two methods were counted as overlapping.

### Pathway enrichment analysis

We used ShinyGO with chicken as background to perform pathway enrichment analysis and identify genes that are under selection in specific agroecologies [108].

### Association analyses

Association analyses in RDA were performed with the R package ‘vegan’ using RDA function [109] to identify environmental predictors and quantitative traits associated with genomic variation. RDA is a multivariate multiple regression followed by a PCA of the table of fitted values and presents relationships between variables in two-dimensional space using ordination plots [80, 110, 111]. Environmental predictors and quantitative traits were analysed separately according to [112].

RDA was performed using a set of genome-wide LD-pruned SNPs to keep a subset of SNPs that are nearly uncorrelated with each other and keep a subset of markers that are in approximate linkage equilibrium. Pruning of genotypes for high LD reduces redundant loci and improves efficiency of models in association analysis [113]. The cleaned dataset with 25 M SNPs filtered for SNPs not in Hardy-Weinberg equilibrium (*p* < 5 × 10^− 6^) and MAF < 5%, was LD pruned using PLINK [114]. We used the following setting for pruning: plink2 --vcf --set-all-var-ids @:# --chr-set 38 --allow-extra-chr --indep-pairwise 100 10 0.5 --maf 0.05 --recode vcf-iid --out --indep-pairwise. The ‘100 10 0.5’ instructs PLINK to perform LD pruning by evaluating LD in sliding windows of 100 variants, removing variants within each window if more than 10 are correlated, and considering variants to be correlated if their LD correlation (*r*^*2*^) exceeds 0.5. The ‘--recode vcf-iid’ modifier produces sample IDs in the last header row of VCF file. The LD pruning resulted in a subset of markers comprising 1,070,305 SNPs from 466 individuals, which was large enough for RDA. The dataset was structured as a matrix of 466 chickens by ~ 1 million SNP markers.

### Genotype-environment association (GEA) analysis with RDA

Correlated predictors cause problems for regression-based models like RDA and variable reduction was done when correlation coefficients between ecological predictors exceeded and an acceptable threshold (*r* >|0.7|) [115]. We fitted partial RDA with the 10 selected environmental predictors conditioned on (i.e., controlling for the effects of) geography as explanatory variables and the genetic dataset as response variable [81]. SNPs exhibiting RDA loadings greater than 3.5 standard deviations (two-tailed *p*-value = 0.0005) from the mean were identified as selection signals This threshold is very conservative and helps to identify loci under strong selection (i.e., minimizes false positive rates) [59]. After a visual inspection of the scree plots, we extracted SNP loadings from the first three canonical axes.

### Genotype-phenotype association analysis with RDA

We fitted partial RDA with the five least correlated and most explanatory quantitative traits selected by correlation analysis. The RDA were fitted with the quantitative traits as explanatory variables, conditioned on geography, and the genetic dataset as response variable. SNPs exhibiting RDA loadings greater than three and half standard deviations from the mean were identified as association signals [59]. After a visual inspection of the scree plots, we extracted SNP loadings from the first three canonical axes.

## Results

### Habitat suitability

**Fig. 3:**
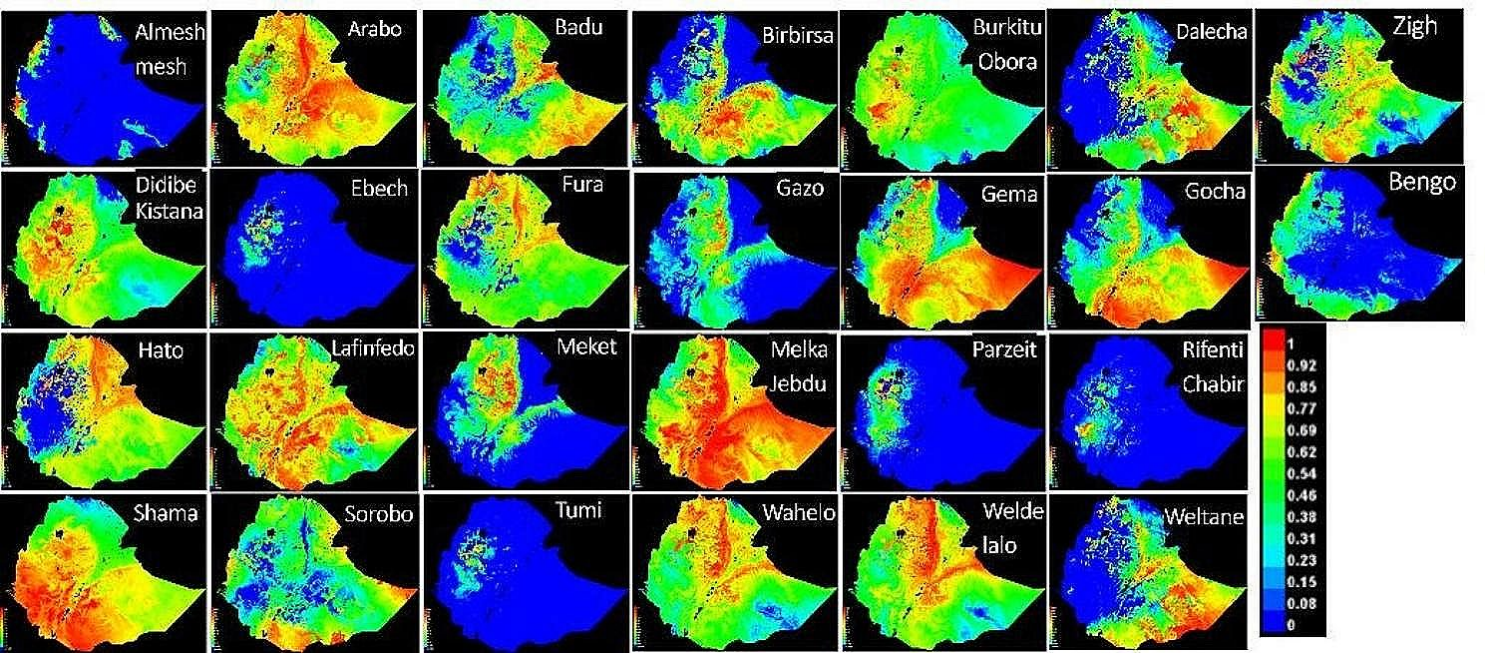
Habitat suitability maps of the 26 Ethiopian chicken populations. Colours towards red spectrum indicate more suitable conditions

The suitability of an environmental niche for a population depends on which environmental predictors are influencing the species. The habitat suitability maps produced by SDMs suggests that the 26 populations have different niches (Fig. 3).

### Genomic diversity of Ethiopian indigenous chickens

**Fig. 4:**
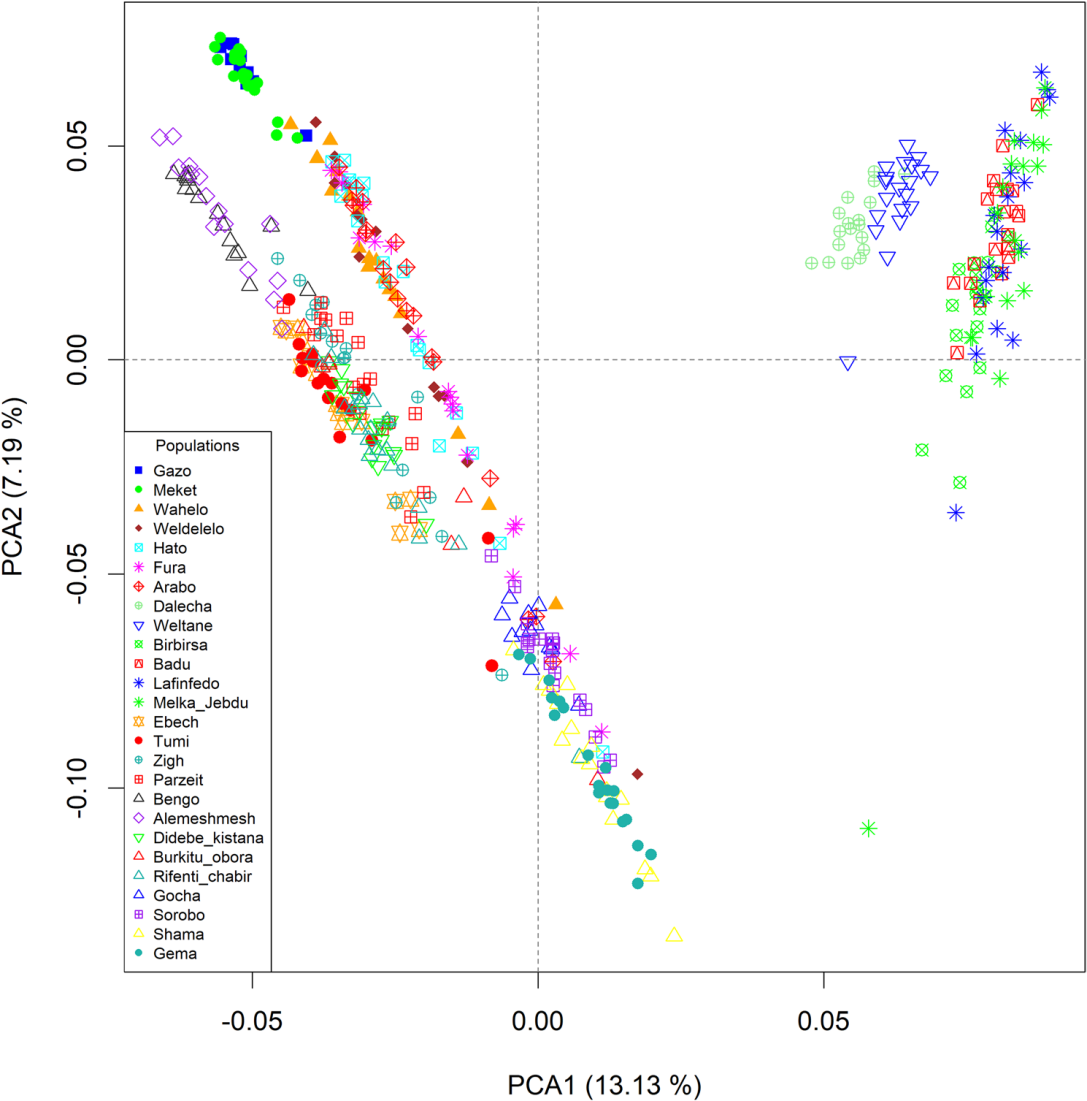
PCA plots of 26 Ethiopian indigenous chickens by population based on 25 million autosomal SNPs

**Fig. 5:**
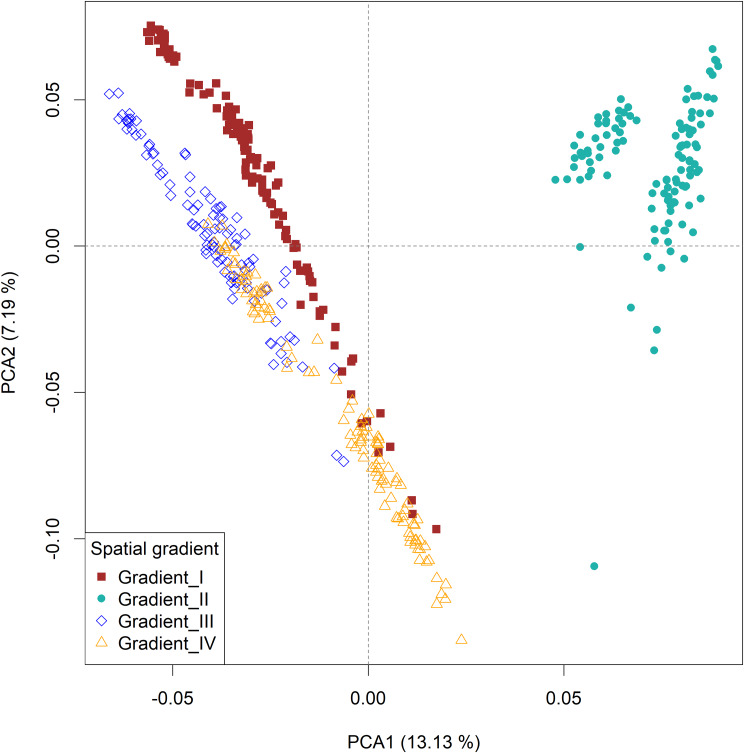
PCA plots of 26 Ethiopian indigenous chickens by gradient based on 25 million autosomal SNPs

**Fig. 6:**
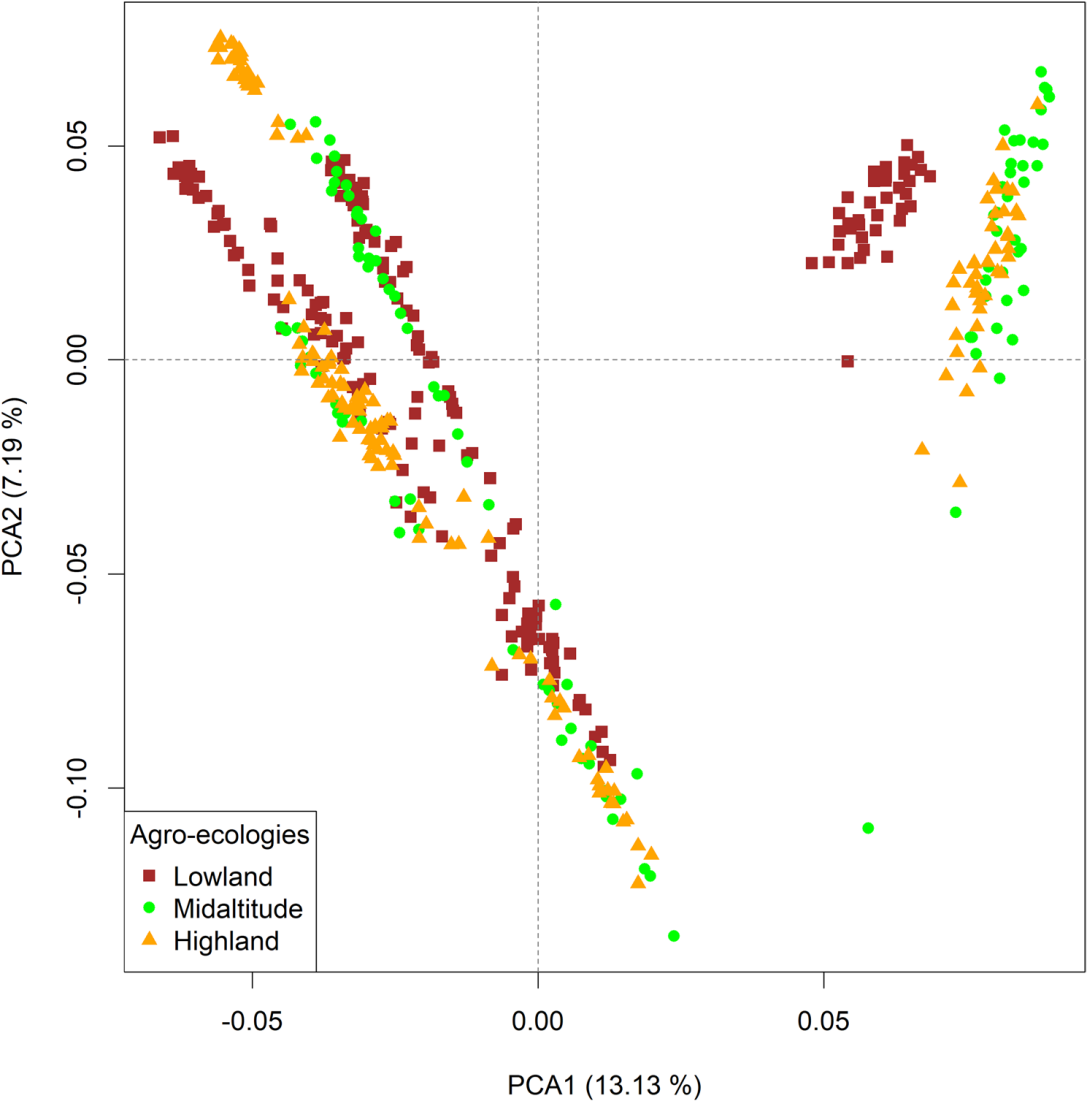
PCA plots of 26 Ethiopian indigenous chickens by agroecology based on 25 million autosomal SNPs

PCA based on the filtered variants provides information on the structure and relatedness of the 26 Ethiopian indigenous chicken sample populations (Figs. 4, 5 and 6). The PCA shows no clear separation among populations (*n = 20*) sampled from *gradient-I, III*, and *gradient-IV*, while populations (*n = 6*) sampled from *gradient-II* have distinctly separated from the other three gradients (Fig. 4).

The PCA based on gradients (Fig. 5**)** illustrates clear separation between chickens sampled from *gradient-II* and the other three gradients. Admixture is seen between *gradients-I* and -*IV*, and between *gradients-III* and *-IV*.

Chicken populations sampled from the three agroecologies (lowland, midlatitude, and highland) did not clearly differentiate except in *gradient-II* where populations sampled from the lowlands were distinct from those sampled from the midlands and highlands of the same gradient (Fig. 6**).**

After carefully looking at the three PCA plots and understanding the genetic structure (Figs. 4, 5 and 6**)** of Ethiopian chickens, we decided that the sampled populations from *gradient-II* should not be analysed together with populations from the other three gradients.

### Signatures of selection for environmental adaptation

#### Genetic differentiation between gradients

The mean $$ { F}_{ST }$$values between any two gradients was low (Table 1). This suggests that genetic differentiation between geographies among Ethiopian indigenous chicken populations is very little. A complete list of significant genes (*p* < 0.01) from overlapping windows jointly identified by $$ { F}_{ST }$$ and XP-EHH in each gradient-wise comparisons is presented in Supplementary Table 5.

**Table 1:** Mean$$ { F}_{ST }$$ scores between chicken populations sampled from different gradients

Between gradients *(layer-I)*	
	Mean $$ { F}_{ST }$$
*I vs. III*	0.021
*I vs. IV*	0.028
*III vs. IV*	0.021

The Manhattan plots of $$ { F}_{ST }$$analyses show pairwise comparison between populations sampled from environmental *gradients-I, -III*, and *-IV* (Supplementary Fig. 1). Some regions of the genome show genetic differentiation across gradients.

### Genetic differentiation between agroecologies across gradients

The mean $$ { F}_{ST }$$values between any two agroecologies across gradients was lower than values obtained for comparisons between any two gradients **(**Table 2**)**. The Manhattan plots on $$ { F}_{ST }$$and their scores for comparisons across agroecologies (Supplementary Fig. 2; Supplementary Table 6) show very low values across the genome. The $$ { F}_{ST }$$scores for comparisons between agroecologies within *gradient-II*(Table 2, Supplementary Fig. 3, Supplementary Table 6) are relatively higher than between agroecologies across gradients, owing to differences in genetic background evidenced by the population structure analysis, Figs. 4, 5 and 6.

**Table 2:** Mean$$ { F}_{ST }$$ scores between chicken populations sampled from different agroecologies

Between agroecologies		
	Mean$$ { F}_{ST }$$ between agroecologies across gradients (*layer-II*)	Mean$$ { F}_{ST }$$ between agroecologies within *gradient-II* (*layer-III*)
Lowland-Midland	0.009	0.021
Lowland-Highland	0.004	0.018
Midland-Highland	0.011	0.009

### Selection signatures between agroecologies across gradients

**Fig. 7:**
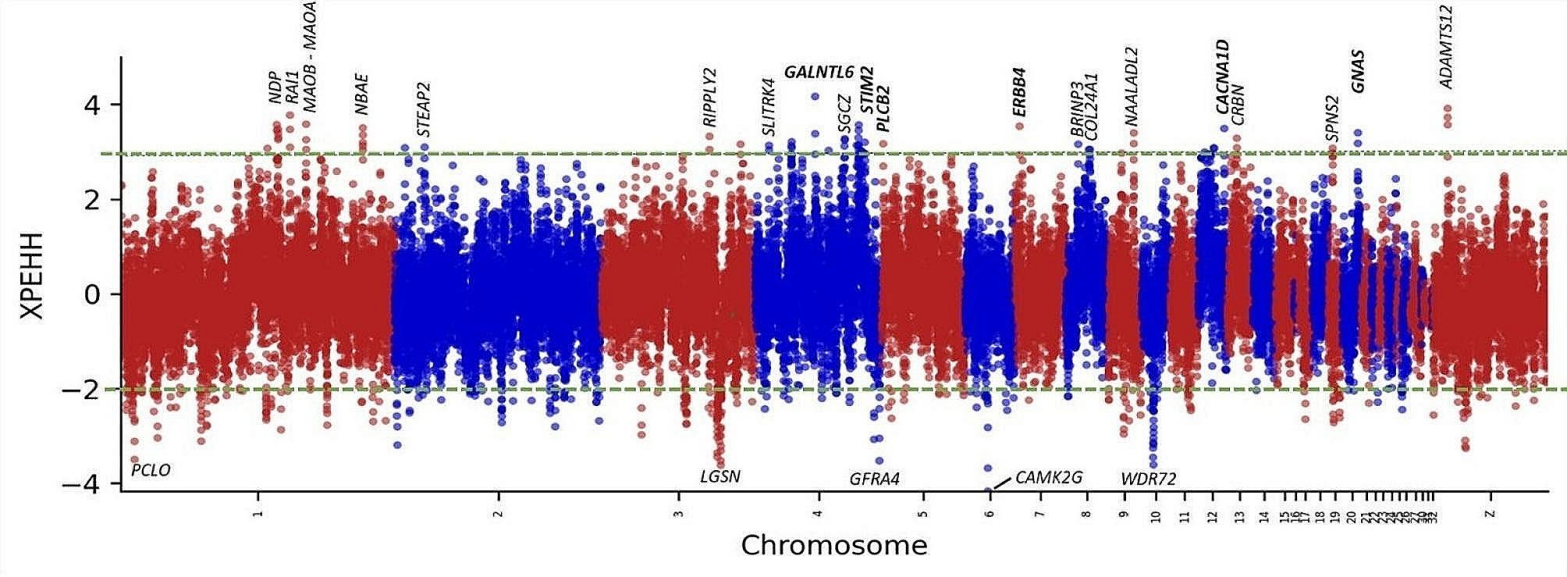
XP-EHH plots for overlapping bins of 50 kb indicating positive selection in the highland populations while negative values indicating selection in lowland populations of Ethiopian indigenous chicken populations sampled across three *gradients* (*-I,-III, and -IV*). All SNPs with a -log (*p*-value) above 2 or below − 2 from the green line are significantly selected (*p* < 0.01) in one agroecology but not in the other. Genes indicated in bold have been associated with the calcium signalling pathway or hypoxia

We performed XP-EHH analysis to identify genomic loci associated with high-altitude adaptation. Figure 7 shows the most significant selective sweeps for highland vs. lowland populations. All SNPs with a -log (*p*-value) above 2 or below − 2 from the green line are significantly selected (*p* < 0.01) in one agroecology but not in the other. The most significant window under selection in the highland populations was found on chromosome four, overlapping the *GALNTL6* gene (XP-EHH = 4.16). Variants in this gene have been associated with power performance in humans [116] possibly by showing a positive effect on anaerobic metabolism. In addition, several genes under selection (XP-EHH > 2.7) are part of the calcium signalling pathway which has been associated with high altitude adaptation and hypoxia in previous studies including Tibetan chickens [19]. The genes identified in this pathway include *ERBB4* [117], *PLCB2*, *STIM2* [118], and *GNAS* [119]. The *MOAA* and *MOAB* genes are also under strong selection in the high altitude populations (XP-EHH > 3.5), these genes correlate with the expression of HiF-1α and with transcription factors Sp1 and Sp3 which are master regulators of the cellular and developmental response to hypoxia [120]. Other genes under selection include the *RIPPLY2*, associated with body length [121], the *SGCZ* gene which response to the HIF-1 transcription activity during hypoxia [122], the *SPNS2* gene that regulated hypoxia-inducible factor 2alpha [123], and the *BRINP3* gene under selection in high-altitude Andeans [124]. A complete list of genes from overlapping windows jointly identified by $$ {F}_{ST}$$ and XP-EHH in agroecological comparisons in lowland vs. highland, lowland vs. midland, and midland vs. highland respectively across the three gradients (*layer-II*) are presented respectively in Supplementary Table 7. XP-EHH scores for comparisons between different agroecologies across the three gradients (*layer-II*) and between gradients are presented in Supplementary Tables 8 and Supplementary Table 9 respectively.

### Selection signatures between agroecologies within *gradient-II* (*analytical layer-III*)

**Fig. 8:**
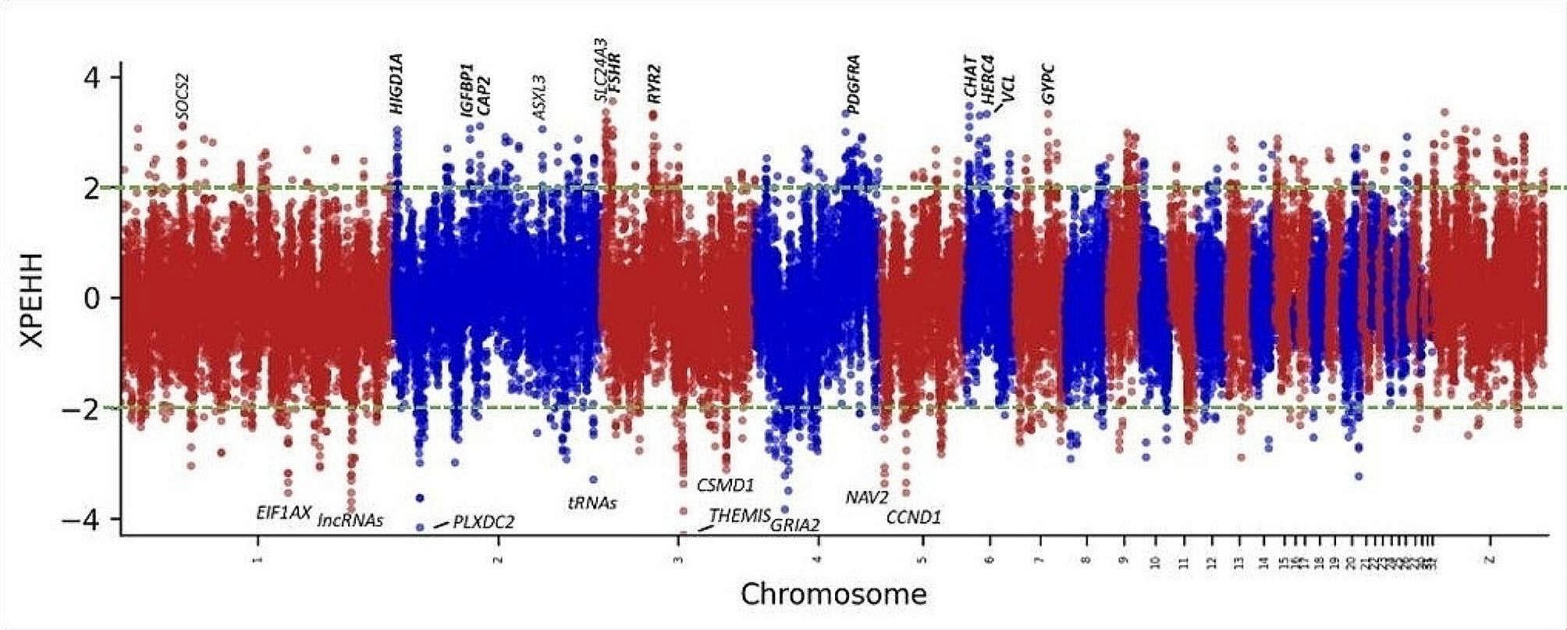
XP-EHH plot for highland vs. lowland in *gradient-II*. All SNPs with a -log (*p*-value) above 2 or below − 2 from the green line are significantly selected (*p* < 0.01) in one agroecology but not in the other. Positive XP-EHH values indicate positive selection in the highland populations while negative values indicate selection in lowland populations. Genes indicated in bold have been associated with the hypoxia related pathways

Figure 8 shows the most significant selective sweeps for highland vs. lowland populations within *gradient-II*. The most significant window was found on chromosome 3 overlapping the follicle stimulating hormone receptor (*FSHR*) gene, which is an activator of the hypoxia-inducible factor-1 protein [125], a key regulator of oxygen homeostasis. The second strongest signal is found on chromosome 6 overlapping the *CHAT* gene which has a direct interaction with the hypoxia-inducible factor (HIF)-1α protein [126]. Interestingly, the third strongest peak overlaps with the *RYR2* gene. This gene is well known to be associated with high altitude adaptation in Tibetan chickens [19]. Other notable genes under selection include *HIGD1A* (hypoxia inducible domain family, member 1 A), *IGFBP1* (insulin like growth factor), *CAP2*, and *HERC4*, of which the latter two have been found to be differentially expressed under hypoxic environments [18].

There is no significant enrichment for the highland genes found. However, the genes under selection in the lowland populations are enriched for the ECM-receptor interaction and focal adhesion which serve as an important role in tissue and organ morphogenesis and in the maintenance of cell and tissue structure and function [127].

A significant overlap (13.4%) was observed between significant windows (*p* < 0.01) identified by $$ {F}_{ST }$$and XP-EHH analyses in the pairwise agroecological comparisons across gradients and within *gradient-II* (20.9%), indicating that the two methods target the same regions and hence are good predictors of selection signatures. Additional details are given under Supplementary Fig. 5 for overlaps between the two methods across gradients and under Supplementary Fig. 6 for overlaps within *gradient-II*. Complete list of genes from overlapping windows jointly identified by $$ { F}_{ST }$$ and XP-EHH in agroecological comparisons across gradients and within *gradient-II* are also presented in Supplementary Table 10 A–C and Supplementary Table 10D–F, respectively.

### Pathway enrichment analysis

We assessed whether the genes under selection in the highland population are enriched for specific KEGG pathways. In total 150 bins have a XP-EHH value greater than 2.7 (*p < 0.01)*. These bins include a total of 95 genes. Only the “Calcium signalling pathway” was significantly enriched (q-value < 0.1) and in which five genes were found (*ERBB4, GRIN2A, STIM2, GNAS, PLCB2*) to be under selection in the highland populations. Several candidate genes in the calcium-signalling pathway were found to be under directional selection in adaptation to the hypoxia experienced by two Tibetan chicken populations [19], suggesting a potential genetic mechanism underlying high altitude adaptation to be similar in Ethiopian highland chicken compared to Tibetan chickens [19]. Ca^2+^ are signalling molecules that regulate the response to hypoxia, which modulates cell contraction, cell proliferation and growth [128, 129]. Moreover, calcium signalling stimulates the translation of HIF-alpha, a transcription factors that mediates adaptation to hypoxia [130]. The candidate selected genes identified in this study, and their variants, may be useful targets for clarifying our understanding of high-altitude adaptation in chicken. In addition, the “ribosome pathways” is enriched for windows under selection in the lowland populations (XP-EHH < -2) from a total of 212 windows and 112 genes.

XP-EHH detected signatures of selection between populations sampled from any two gradients were also strong (Supplementary Fig. 4.). However, they were not as strong as signatures detected across agroecologies.

### Pathway enrichment analysis

We assessed whether the genes under selection in the highland population are enriched for specific KEGG pathways. In total 150 bins have a XP-EHH value greater than 2.7 (*p < 0.01)*. These bins include a total of 95 genes. Only the “Calcium signalling pathway” was significantly enriched (q-value < 0.1) in which five genes were found (*ERBB4, GRIN2A, STIM2, GNAS, PLCB2*) to be under selection in the highland populations. Several candidate genes in the calcium-signalling pathway were found to be under directional selection in adaptation to the hypoxia experienced by two Tibetan chicken populations [19], suggesting a potential genetic mechanism underlying high altitude adaptation to be similar in Ethiopian highland chicken compared to Tibetan chickens [19]. Ca^2+^ are signalling molecules that regulate the response to hypoxia, which modulates cell contraction, cell proliferation and growth [128, 129]. Moreover, calcium signalling stimulates the translation of HIF-alpha, a transcription factors that mediates adaptation to hypoxia [130]. The candidate selected genes identified in this study, and their variants, may be useful targets for clarifying our understanding of high-altitude adaptation in chicken. In addition, the “ribosome pathways” is enriched for windows under selection in the lowland populations (XP-EHH < -2) from a total of 212 windows and 112 genes.

XP-EHH detected signatures of selection between populations sampled from any two gradients were also strong (Supplementary Fig. 4). However, they were not as strong as signatures detected across agroecologies.

#### Genotype-environment associations (GEA)

Of the ten predictors identified through MaxEnt-based SDMs for their association with habitat suitability of chickens [46] and elevation (added as a tenth predictor), 6 less correlated (*r*≤|0.7|) predictors were retained for RDA (Supplementary Fig. 5). These predictors were: precipitation of the warmest quarter, precipitation of the coldest quarter, solar radiation of May, elevation, soil clay content and temperature seasonality. We had as many RDA axes as we had predictors (n  = 6) in our model. The first three RDA axes explained more than half (68.1%) of the variance in the environmental predictors (Supplementary Table 11). The significance of models in RDA is based on *F*-statistics [131].The adjusted *R*^*2*^ considering the number of environmental predictors was 0.02, meaning that our constrained ordination explains about 2% of the variation or that 2% of the SNP variation is associated with the environmental predictors. Based on the magnitude of the arrows in PCA plots based on RDA axes 1 and 2 (Supplementary Fig. 8) elevation, precipitation of the warmest quarter, and soil clay content had the highest contributions to genotypic variation, while temperature seasonality and solar radiation had the lowest contributions.

**Fig. 9:**
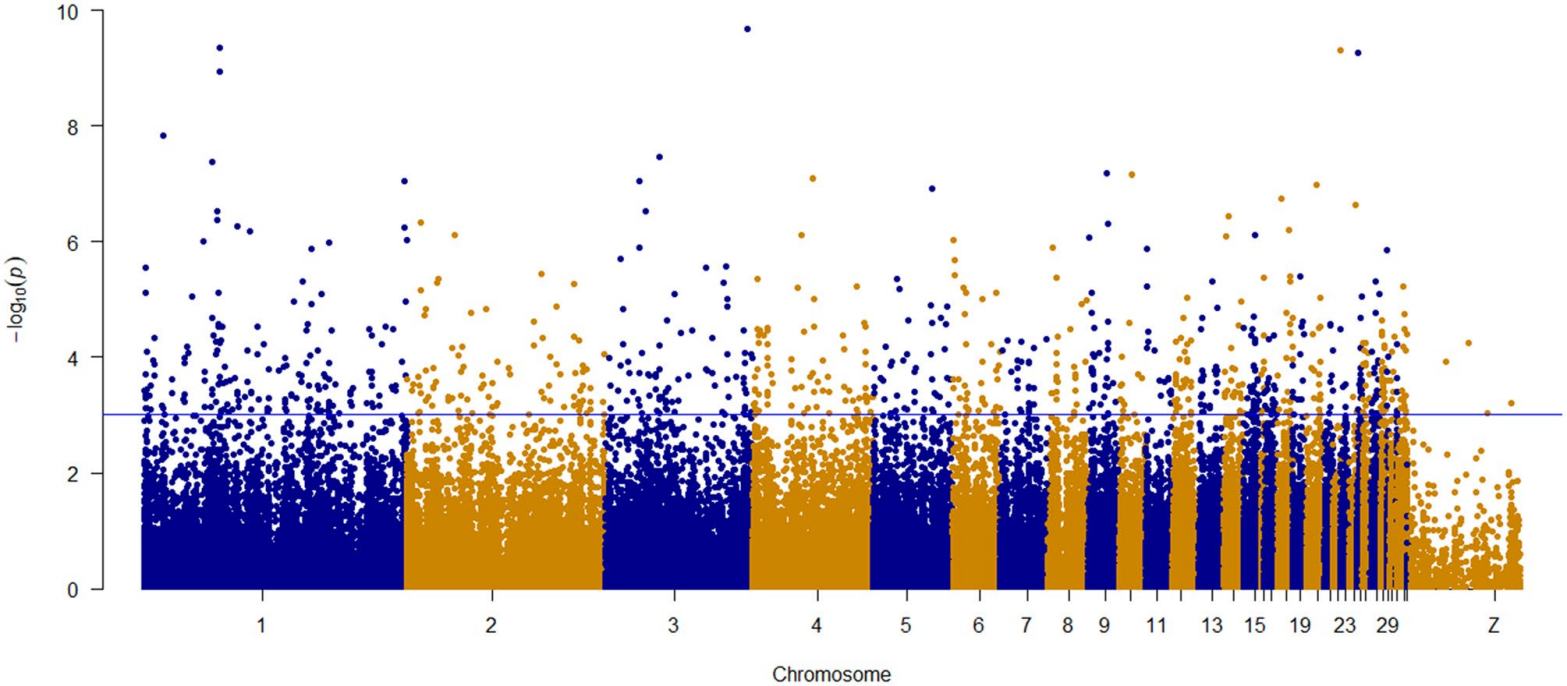
Manhattan plot of RDA showing the association of SNPs with the combined set of six environmental predictors in the three *gradients* (*-I, -III*, and *-IV*) as explanatory variables. The y-axis indicates -log 10 (*p-*value). Horizontal blue line indicates the significance threshold (*p* < 0.001)

The SNP loadings for environmental predictors on each of the three RDA axes show a relatively normal distribution (Supplementary Fig. 9). The 1,909 SNPs from the two extreme ends of the loading distribution with standard deviation > 3.5 (two-tailed *p*-value = 0.0005) for each significant axis were taken as outlier SNPs that are associated with environmental variation. The list of candidate SNPs which have significant association (*p* < 0.001) with the six environmental predictors and considered to be under selection are presented in Supplementary Table 12. SNPs associated with the combined set of environmental predictors in *gradients -I, III, and -IV* do not show a clear clustering but are more or less evenly spread across the genome (Fig. 9).

**Fig. 10:**
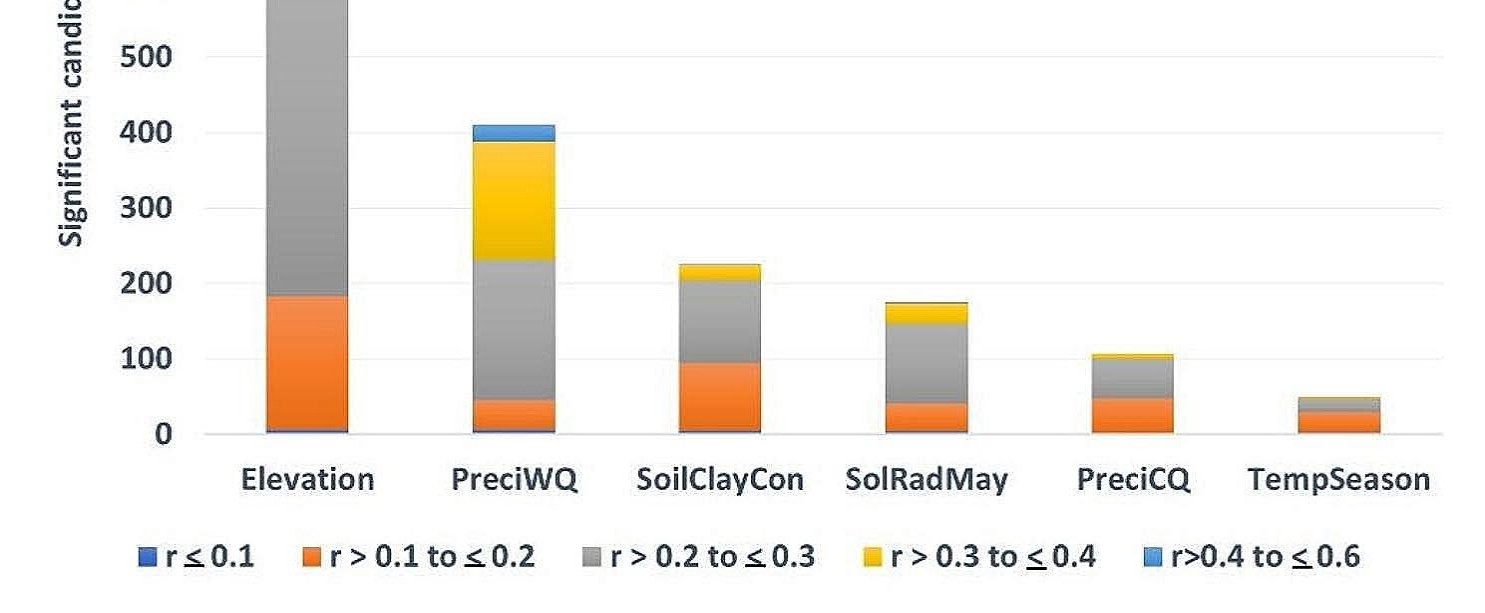
Number of significant candidate SNPs (*p* < 0.001) that are most correlated with each of the six selected environmental predictors grouped by absolute magnitude of their correlation

Some of the highest -log10 (*p*-values) are found on chromosomes 1 and 3 (Fig. 9). Only the peak on chromosome 1 shows additional significant SNPs near the top SNP. The significant candidate SNPs (*n* = 1,909) that are associated with the combined set of environmental predictors are assigned to individual predictors based on the correlation values estimated by partial RDA analysis (Fig. 10). Most candidate SNPs (942 or 49.3%) have their highest correlation with elevation. Elevation has also the highest number (*n* = 321 or 57.4%) of the moderately to highly associated SNPs (*n* = 559) (0.3 < *r* < 0.6). The second environmental predictor most associated with candidate SNPs is precipitation of the warmest quarter. It has correlation with 410 candidate SNPs (21.47%). The other 4 environmental predictors have the highest correlation for a smaller number of SNPs (*n* = 557 or 29.17%), but for all predictors, a considerable number of SNPs (*n* = 59) are found with correlations above|0.3| and only two SNPs have correlations above|0.4|.

### Genotype-phenotype association

Out of a total of 8 phenotypic variables identified through MaxEnt-based SDMs for their utility in phenotypically discriminating study populations [46], five least correlated (|*r*| ≤ 0.72) quantitative traits were selected to be used for RDA (Supplementary Fig. 10). These five traits were live body weight, beak length, comb width, wattle width and earlobe width. The correlation between comb width and wattle width was 0.72 which is slightly higher than the common threshold (|*r*| > 0.7) used to reduce variables However, we decided to keep both traits because of their adaptive roles documented in literature related with thermoregulation in tropical chickens. The first three RDA axes explained most of the variance (62.1%) in the phenotypic predictors (Supplementary Table 13). The adjusted *R*^*2*^ for the partial RDA was 0.002. This shows that only 0.2% of the SNPs variation is associated with quantitative traits.

**Fig. 11:**
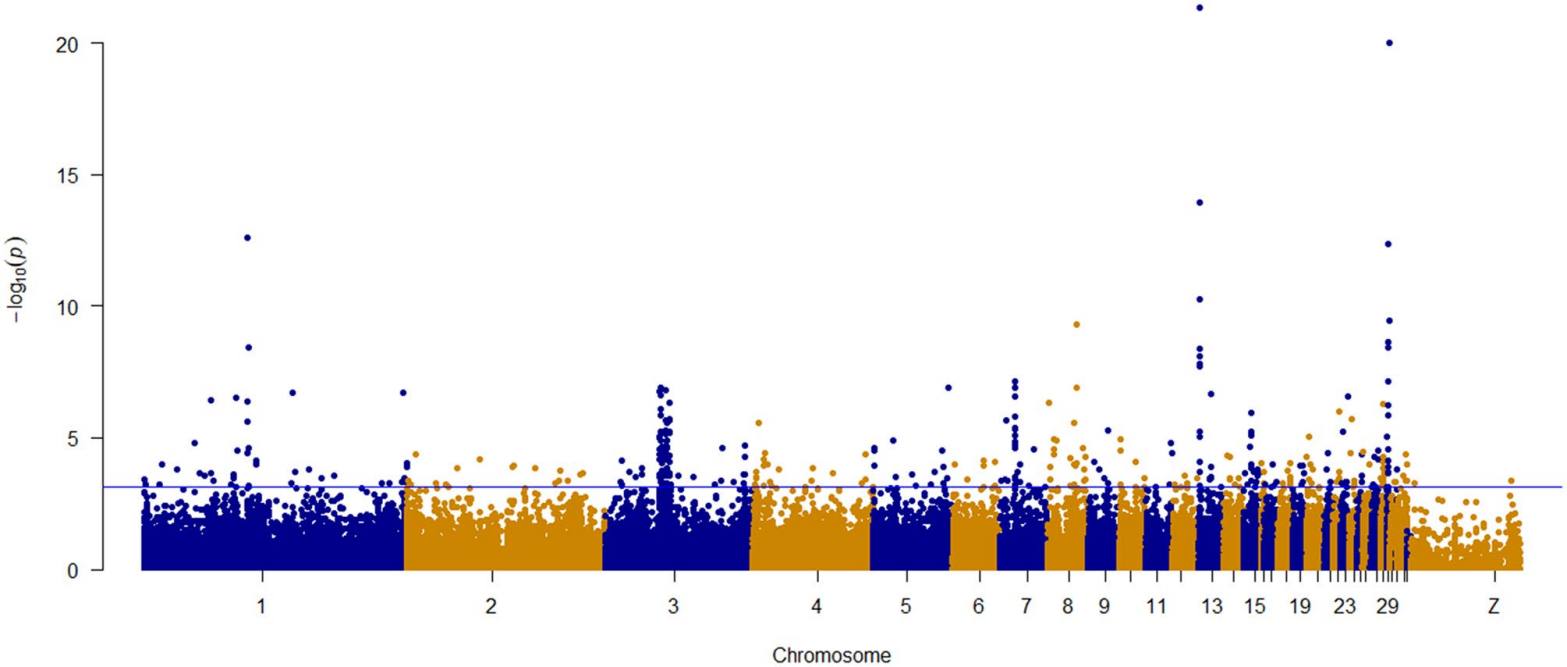
Manhattan plot of RDA showing the association of SNPs with phenotypic variation in the five quantitative traits in *gradients -I, -III*, and*-IV*. The y-axis indicates -log 10 (p-value). Horizontal blue line indicates the significance threshold (*p* < 0.001)

The SNP loadings for quantitative traits on each of the three RDA axes show a relatively normal distribution (Supplementary Fig. 11). Based on the magnitude of the arrows in the PCA plots based on RDA axes 1 and 2 (Supplementary Fig. 12), comb width, wattle width and body weight were most useful in explaining SNP variation. SNPs associated with the combined set of quantitative traits in *gradients -I, -III*, and -*IV* show strong supportive peaks on chromosomes 1,3, 4, 7,8, 13, 15, and 29 indicating probable regions of quantitative trait loci (QTL) associated with phenotypic variation (Fig. 11). The picks were more diffused across the genome for the Manhattan plot showing association between SNPs and quantitative traits for populations sampled from *gradient-II* (Supplementary Fig. 13).

**Fig. 12:**
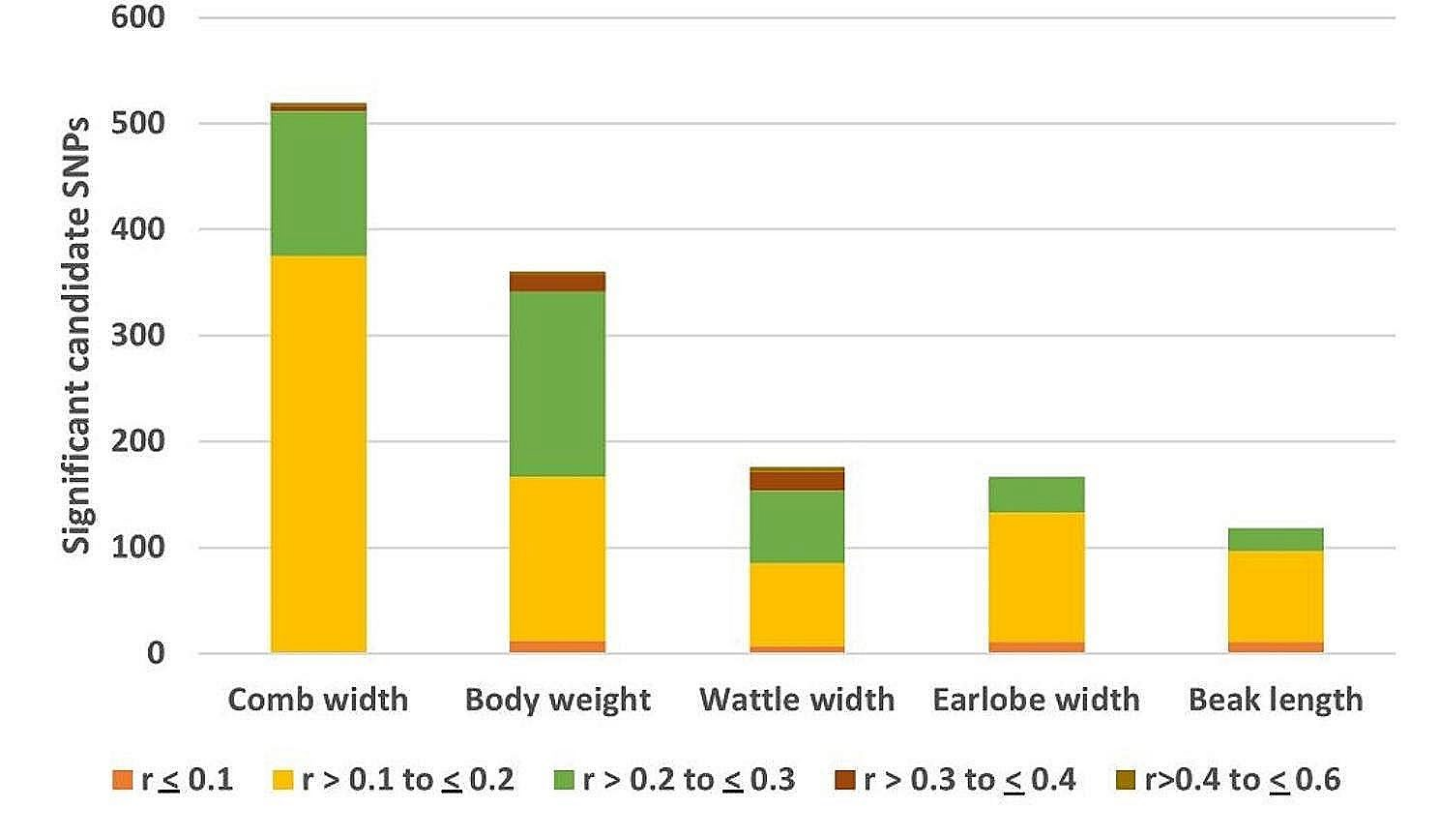
Number of significant candidate SNPs (*p* < 0.001) that are most correlated with each of the five quantitative traits, grouped by absolute magnitude of their correlation

A stacked bar chart showing the number of outlier SNPs (*p* < 0.001) that are most correlated with each of the five quantitative traits is presented in Fig. 12. The significant candidate SNPs (were assigned to individual traits based on correlation values estimated by partial RDA analysis. Partial RDA identified 1340 candidate SNPs that have significant association with the five quantitative traits (Supplementary Table 14). A total of 19 SNPs show moderate to high correlation with body weight (0.3 < *r* ≤ 0.6). Most candidate SNPs, 39%, were associated with comb width (*n* = 519) and 27% with body weight (*n* = 360) **(**Fig. 12 and Supplementary Table 13. Higher association was also seen between comb width and candidate SNPs for populations sampled from *gradient-II* (Supplementary Fig. 14).

The list of significant (*p* < 0.001) candidate SNPs identified by RDA and their respective $$ { F}_{ST}$$and XP-EHH values across gradients are presented in Supplementary Tables 15 and in Supplementary Table 16.

## Discussion

Adverse effects of climate change and increasing demand for animal source proteins, particularly in the tropics (particularly in Africa and Southeast Asia), necessitate that we properly understand the genetic architecture of environmental adaptation and develop productive and environmentally resilient breeds [132, 133]. Investigation of molecular pathways indicate that indigenous chickens are more adapted to the environment in which they live compared to specialized chickens [132]. Important insights were obtained from earlier studies on local adaptation of African chickens [45, 134, 135] by applying SDMs and signatures of selection analyses. However, previous studies did not adequately relate genomic variation with environmental and phenotypic variation. Analysing genomic data without relating it environmental and phenotypic variation does not provide a complete picture of adaptive variation.

In the present study, we followed a landscape genomic approach to study adaptive and phenotypic variation among Ethiopian chickens. We applied an environmental-gradation approach to survey chicken populations across all possible agroclimatic clines in the country. Our sample size of 513 animals from four environmental gradients was large enough to capture adaptive variation across populations. For species with limited dispersal, sample sizes above 200 units are generally sufficient to detect most adaptive signals in landscape genomics, while in random mating populations this threshold should be increased to 400 units [84]. After applying stringent quality filtration, we had 25 M SNPs (autosomal and non-autosomal) and 466 individuals for downstream genomic analyses. The dataset used in the present study is substantially larger than previous genomic studies on Ethiopian chickens (which sampled a maximum of 225 birds per study) [45, 136, 137].

We combined different techniques including SDMs, genetic differentiation test ($$ {F}_{ST})$$, cross-population Extended Haplotype Homozygosity (XP-EHH), and RDA.

SDMs were used in our study to identify the most relevant environmental predictors influencing habitat suitability for chickens. The environmental predictors identified in the present study (related with elevation, precipitation, soil clay content, solar radiation, and temperature) were reported in earlier studies for their influences on availability of feed, productivity, prevalence of diseases and parasites [26, 46, 138, 139]. The habitat suitability maps produced by SDMs suggest that the 26 Ethiopian indigenous chicken sample populations may have gone through different environmental selective pressures which give rise to phenotypic and genetic differentiation.

The gradients and agroecologies show low differentiation, as evidenced by the low $$ {F}_{ST}$$ values. Lower level of genetic differentiation was also detected among Ethiopian chickens by [45]. In contrast to the $$ { F}_{ST}$$ results, strong signals of selection (*p* < 0.01) were detected by XP-EHH in pairwise agroecological comparisons. XP-EHH results show that selective pressure in Ethiopian chickens is stronger between agroecologies.

A large overlap was observed between significant windows identified by $$ {F}_{ST }$$and XP-EHH analyses, suggesting that both methods identified similar regions in the genome are under selection. The overlap between$$ { F}_{ST }$$and XP-EHH analyses ranged from 13.4 to 20.9% between agroecologies which is considerably higher than the 4.9% overlap reported by [45] between$$ { F}_{ST }$$and XP-EHH for Ethiopian chickens. The large overlap between $$ {F}_{ST }$$and XP-EHH in the present study might be due to our sampling strategy. Firstly, the sampling design captured a wide range of geographic and environmental variation and helped to survey most of the ecotypes and agroecologies in the country. Secondly, the design may have minimized confounding between neutral and adaptive processes which result from mixing of populations that have different demographic histories. By classifying the populations by gradients, we controlled for the effects of population genetic structure associated with specific geographies. For instance, a very high overlap between the$$ { F}_{ST }$$and XP-EHH results was found in agroecological comparisons within *gradient-II*. The decision to analyse this gradient on its own was informed by PCA, which clearly separated populations of *gradient-II* from the other three *gradients* (*-I*, *-II*, and *-III*). *Gradient-II* represents chicken populations from eastern parts of Ethiopia which have a distinct evolutionary history and route of introduction into the country [38, 40, 136], in contrast to populations representing the other three gradients. Combining *gradient-II* with the other three would have reduced the overlap of $$ { F}_{ST }$$and XP-EHH results.

Our results based on XP-EHH show that genes in the calcium signalling pathway are under selection in high-altitude adapted Ethiopian chicken populations as well as genes associated with the hypoxia-inducible factor (HIF) transcription factors. The gene under strongest selection is the *GALNTL6* gene associated with sports performance in multiple human studies. It is hypothesized that this gene is expressed in the gut microbiome regarding regulation of short-chain fatty acids and their anti-inflammatory and resynthesis functions causing a positive effect on anaerobic metabolism. The ERBB4 gene, found to be under selection in high altitude Ethiopian chicken populations, is also under selection in human Tibetan populations [117]. *ERBB4* is strongly associated with vascular wall stability, and possibly with the production of erythrocytes and belongs to the epidermal growth factor receptor subfamily. We also identified the *MAOB* and *MAOA* genes to be under selection, where *MAOB* has been shown to be correlated with HiF-1α (tumour grade and hypoxia-inducible transcription factor) [120]. Inhibition of *MAOA* in cells may exert antitumour activity in the treatment of prostate cancer [140]. The roles of *MAOA* and *MAOB* genes in local adaptation of chicken need to be further investigated.

Results from signatures of selection analyses with the two methods ($$ {F}_{ST}$$ and XP-EHH) can be used complementarily with RDA to shed light on the relationship between genomic, phenotypic, and environmental variation in local adaptation studies in indigenous chickens. With RDA, we identified 83 candidate SNPs in regions on chromosomes 1,3, 4, 7,8, 13, 15, and 19 that have a moderate to high correlation (0.3 < *r* < 0.6) with live body weight. Conventional GWAS studies in the past identified body weight associated SNPs and QTLs on chromosomes 1,4, 8, 11, 19 in Chinese, Rwandan, and Ethiopian chicken breeds [27, 141–144]. Our results demonstrate that RDA can be used as an alternative approach to GWAS in random mating, indigenous livestock populations which have sufficiently interacted with the environment.

Candidate SNPs associated with the six SDM-identified environmental predictors contributing to habitat suitability were identified by RDA. The RDA found only 2% of the SNP variation to be associated with the six environmental predictors. This is a small value but not unexpected because most of the SNPs are under neutral and therefore not show a relationship with the environmental predictors. SNPs that do show association with the environmental predictors are likely to be under selection. This selection can be in response to these selected predictors that were used in the model or some other environmental variable that is correlated with these predictors.

Candidate SNPs associated with environmental predictors (Fig. 9) were evenly spread across the genome without obvious overlap with the peaks from genotype-phenotype association Fig. 11). Genotype-phenotype associations had very distinct suggesting that phenotypic variation is present among populations for selection to act on it. The environmental drivers could increase haplotypes related to adaptive phenotypic plasticity and morphological variation in indigenous chickens. The pea-comb, a dominant mutation in chickens, drastically reduces the size of the comb and wattle, decreasing heat loss and making the chicken less susceptible to frost lesions [145]. Histological section analysis of dermal papillary layer has revealed that red earlobes have many more blood vessels and were associated with thinner skin than that of white earlobes [146] indicating the role of earlobes in thermoregulation. The total amount of SNP variation associated with phenotypic variation was only 0.2%, in contrast with 2% of the SNP variation associated with environmental variation. The underlying mechanisms of genotype-phenotype associations are well studied and understood in livestock, but this is not the case for genotype-environment associations. Finding 2% of SNP variation related to environment variation is promising for further investigation of the mechanisms leading to these associations.

In summary, in this manuscript we reported the first study integrating phenotypic, genomic, and environmental information on Ethiopian indigenous chickens. Our findings on genomic and phenotypic variability associated with environmental adaptation (e.g., genes selected in highland populations, genes associated with body weight and ecological variables) are useful in the design of breeding programmes aiming at developing more productive and resilient chicken strains (lines) suitable for smallholder systems in the face of climate crisis. The landscape genomic approach followed in the present study can also be used to study adaptive variation in other random mating indigenous livestock populations that are managed extensively to better understand organismal response to environmental variables and develop better breeding strategies.

### Electronic supplementary material

Below is the link to the electronic supplementary material.

Supplementary Material 1

Supplementary Material 2

Supplementary Material 3

## Data Availability

The datasets analysed during the current study are available from the corresponding author on reasonable request.
