# Supplementary material for: Landscape genomics reveals regions associated with adaptive phenotypic and genetic variation in Ethiopian indigenous chickens
Source: BMC Genomics. 2024 Mar 18;25:284. doi: 10.1186/s12864-024-10193-6 (PMC10946127; doi:10.1186/s12864-024-10193-6)
Supplement: Supplementary file 1 — Supplementary Material 1 [file 12864_2024_10193_MOESM1_ESM.docx]

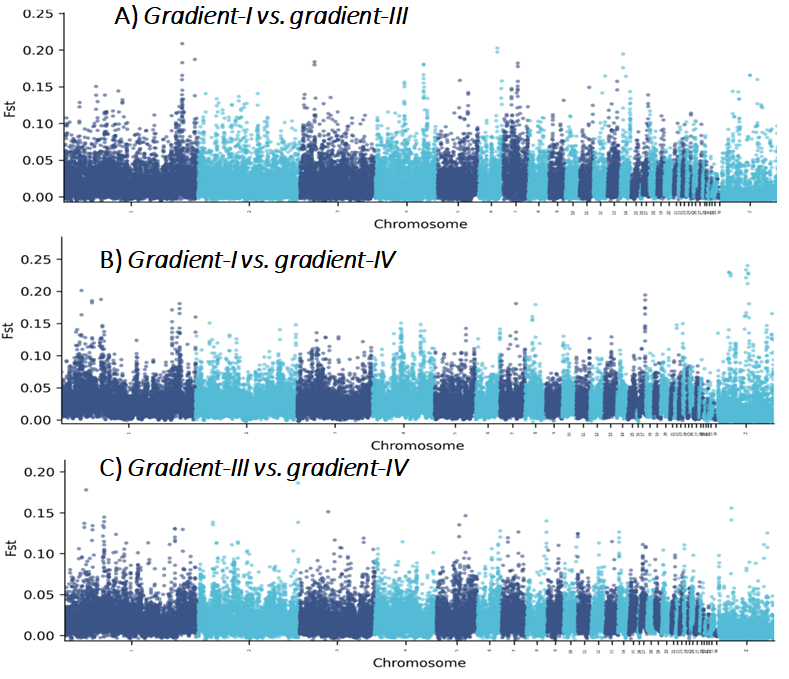


**Supplementary Figure 1.** Manhattan plots of Fixation index (F_ST_) for overlapping bins of 50kb showing pairwise comparisons of Ethiopian indigenous chicken populations between *gradients* (-*I, -III, and -IV)*. **A).** *Gradient-I* vs *gradient-III* **B).** *Gradient-I* vs *gradient-IV*; and **C).** *Gradient-III* vs *gradient-IV*.


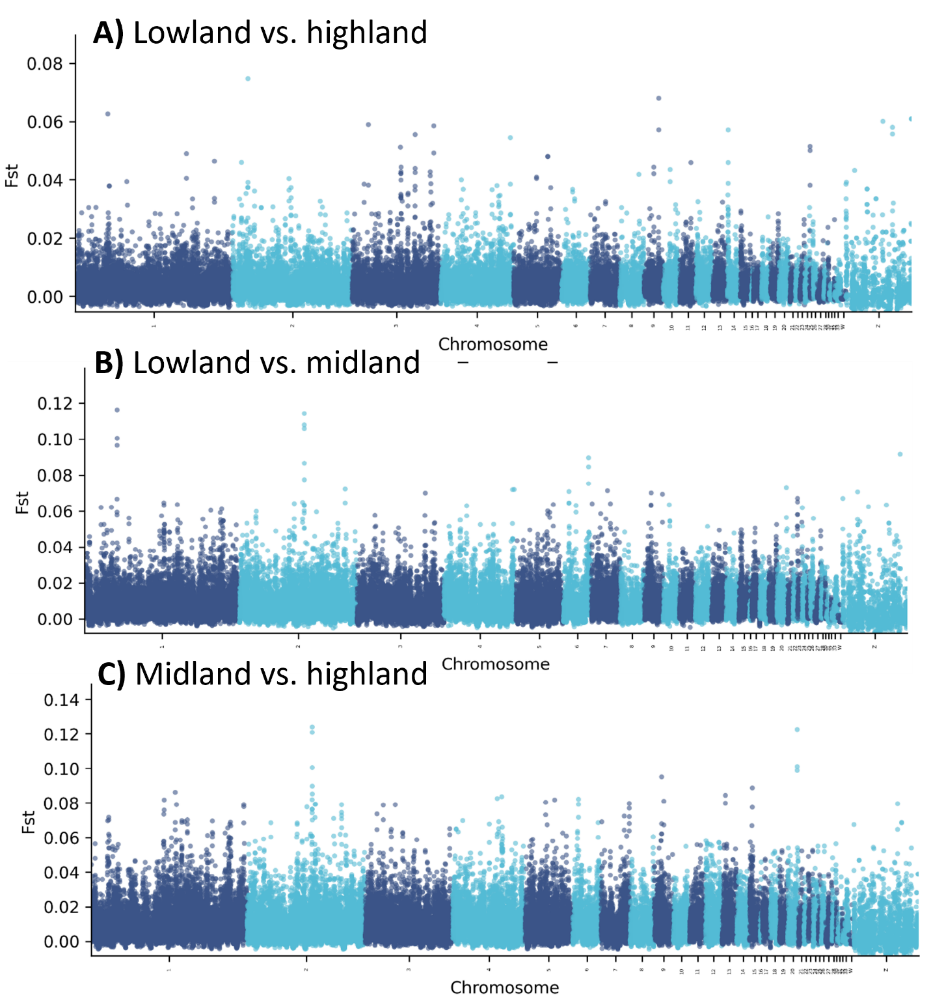


**Supplementary Figure 2.** Manhattan plots of Fixation index (F_ST_) for overlapping bins of 50kb showing pairwise comparison between agroecologies (lowland, midaltitude, highland) for Ethiopian indigenous chicken populations sampled from gradients *-I,-III,* and *-IV* together **A).** Lowland vs highland; **B).** Lowland vs midland; and **C).** Midland vs highland.


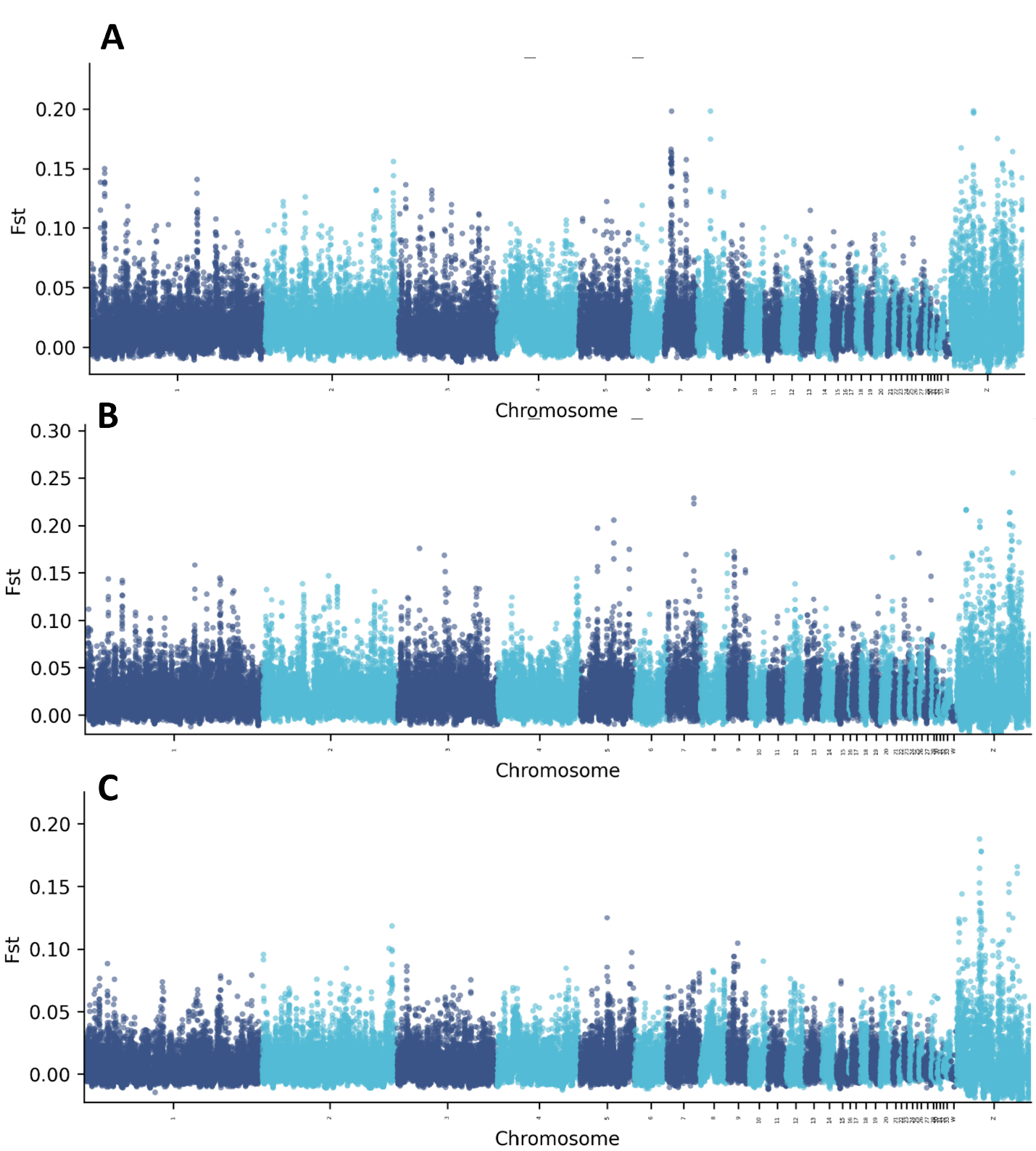


**Supplementary Figure 3.** Manhattan plots of Fixation index (F_ST_) for overlapping bins of 50kb showing pairwise comparison between agroecologies (lowland, midaltitude, highland) for Ethiopian indigenous chicken populations sampled from gradient *-II* **A).** Lowland vs highland; **B).** Lowland vs midland; and **C).** Midland vs highland.

**
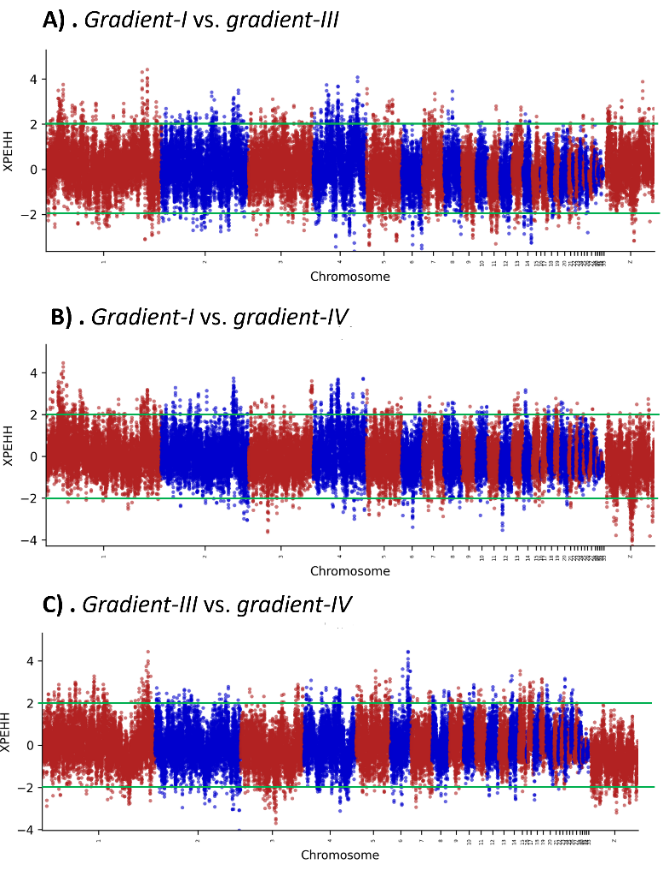
**

**Supplementary Figure 4.** Manhattan plots of XP-EHH for overlapping bins of 50kb showing pairwise comparisons for Ethiopian indigenous chicken populations between *gradients* (-*I, -III, and -IV)*. The y-axis shows -log (p-value), with positive values identifying extended homozygosity in populations from  the first gradient relative to populations from the second gradient, and vice versa for negative values. All SNPs with a -log(p-value) above 2 or below -2 from the green line are significantly selected (p <0.01) in one gradient but not in the other. **A)**. *Gradient-I* vs *gradient-III*; **B)**. *Gradient-I* vs *gradient-IV*; **C)**. *Gradient-III* vs *gradient-IV*.


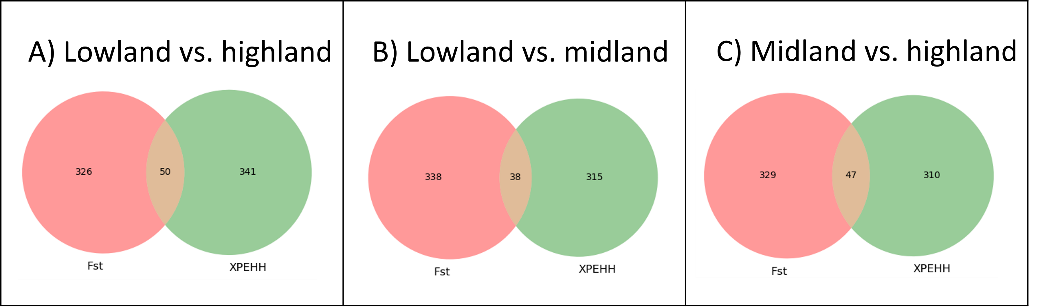


**Supplementary Figure 5.** Venn diagrams showing overlaps between significant (*p* < 0.01) $F_{ST}$ and XP-EHH windows per pairwise comparison between agroecologies across gradients (*-I, -III,* and *-IV*). **A)** Lowland vs highland **B)** Lowland vs midland **C)** Midland vs highland

Higher number of overlapping windows were observed between the two methods of signatures of selection analysis in lowland vs highland and in midland vs highland comparisons compared to lowland vs midland (**Supplementary Figure 5**). A significant overlap (13.4%) was observed between significant windows (*p* < 0.01) identified by $F_{ST}$and XP-EHH analyses in the pairwise agroecological comparisons. There was 14.7% overlap between methods for lowland vs highland, 11.2% overlap between lowland vs midland, and 14.3% overlap between midland vs highland comparison.


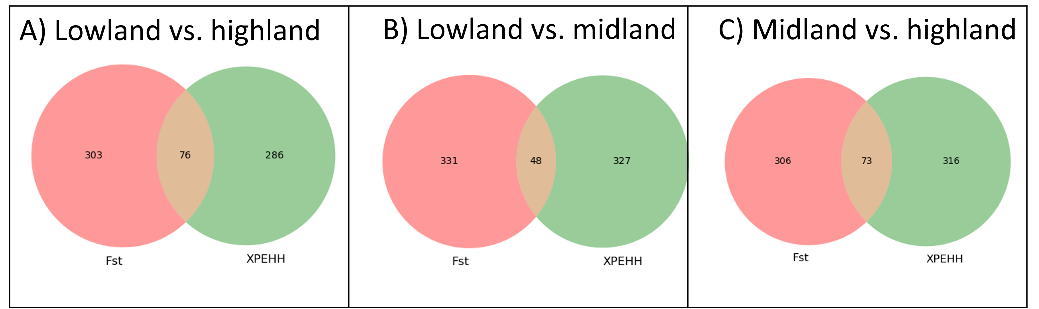


**Supplementary Figure 6.** Venn diagrams showing overlaps between significant (*p* < 0.01) $F_{ST}$ and XP-EHH windows per pairwise comparison between agroecologies within gradient*-II*. **A)** Lowland vs highland **B)** Lowland vs midland **C)** Midland vs highland

The results from agroecological analysis within *gradient-II* show that the overlap between $F_{ST}$ and XPEHH **(Supplementary Figure 6**) increased compared to agroecological analysis across gradients (**Figure 12**). There was 25.1 % overlap between methods for lowland vs highland, 14.5 % overlap between lowland vs midland, and 23.1% overlap between midland vs highland comparison. Selection signatures between agroecologies across gradients were diluted by genetic differentiation associated separate geographies. An overall overlap of 20.9% was observed between significant windows (*p* < 0.01) identified by $F_{ST}$and XP-EHH analyses in the pairwise agroecological comparisons within *gradient-II*.


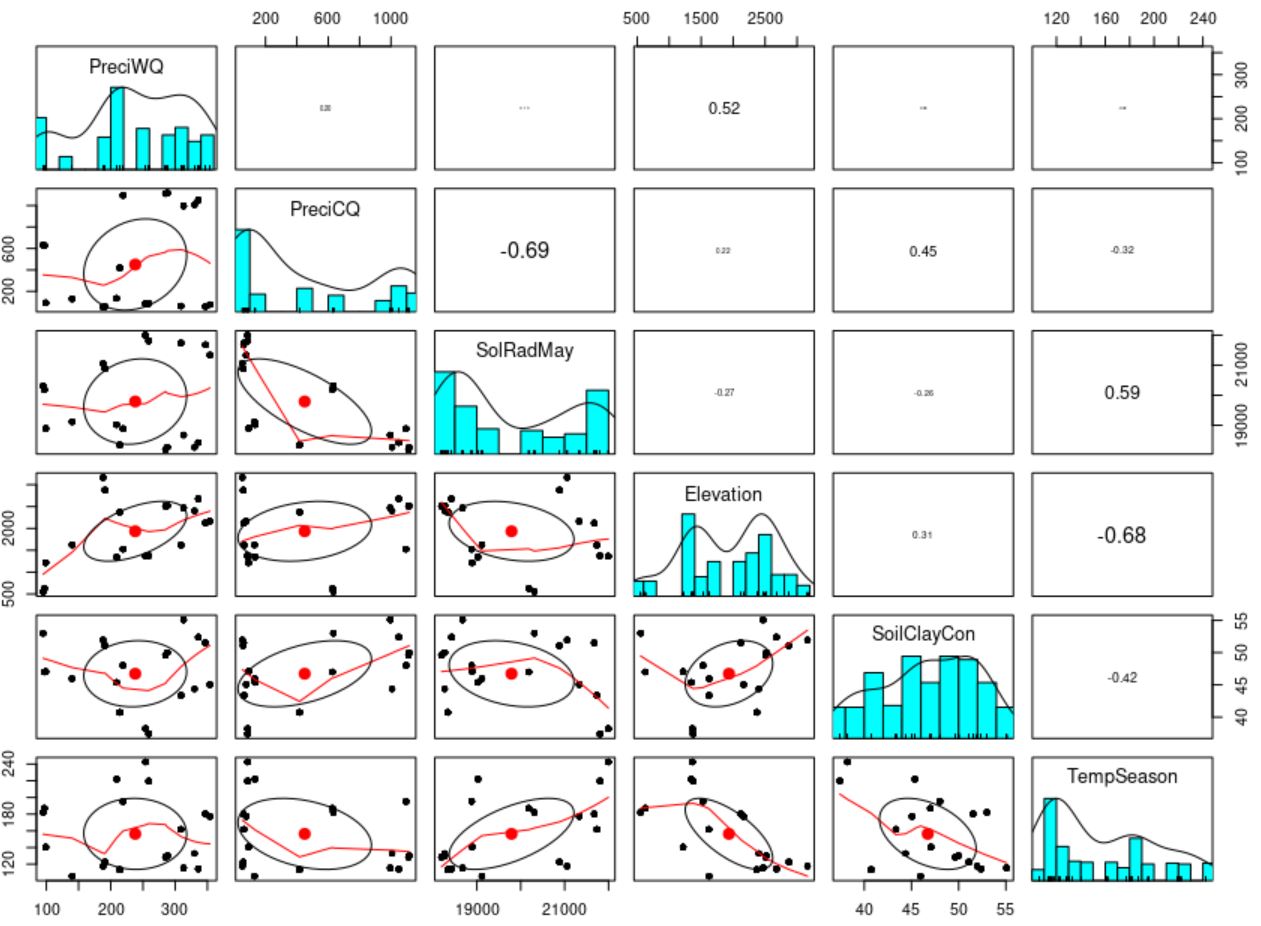


**Supplementary Figure 7..** Correlation among six selected environmental predictors out of nine predictors identified by species and phenotypic distribution models and used for partial RDA. Six predictors were retained to reduce multicollinearity (|r| < 0.7), namely: precipitation of the warmest quarter, precipitation of the coldest quarter, solar radiation of May, elevation, soil clay content and temperature seasonality.


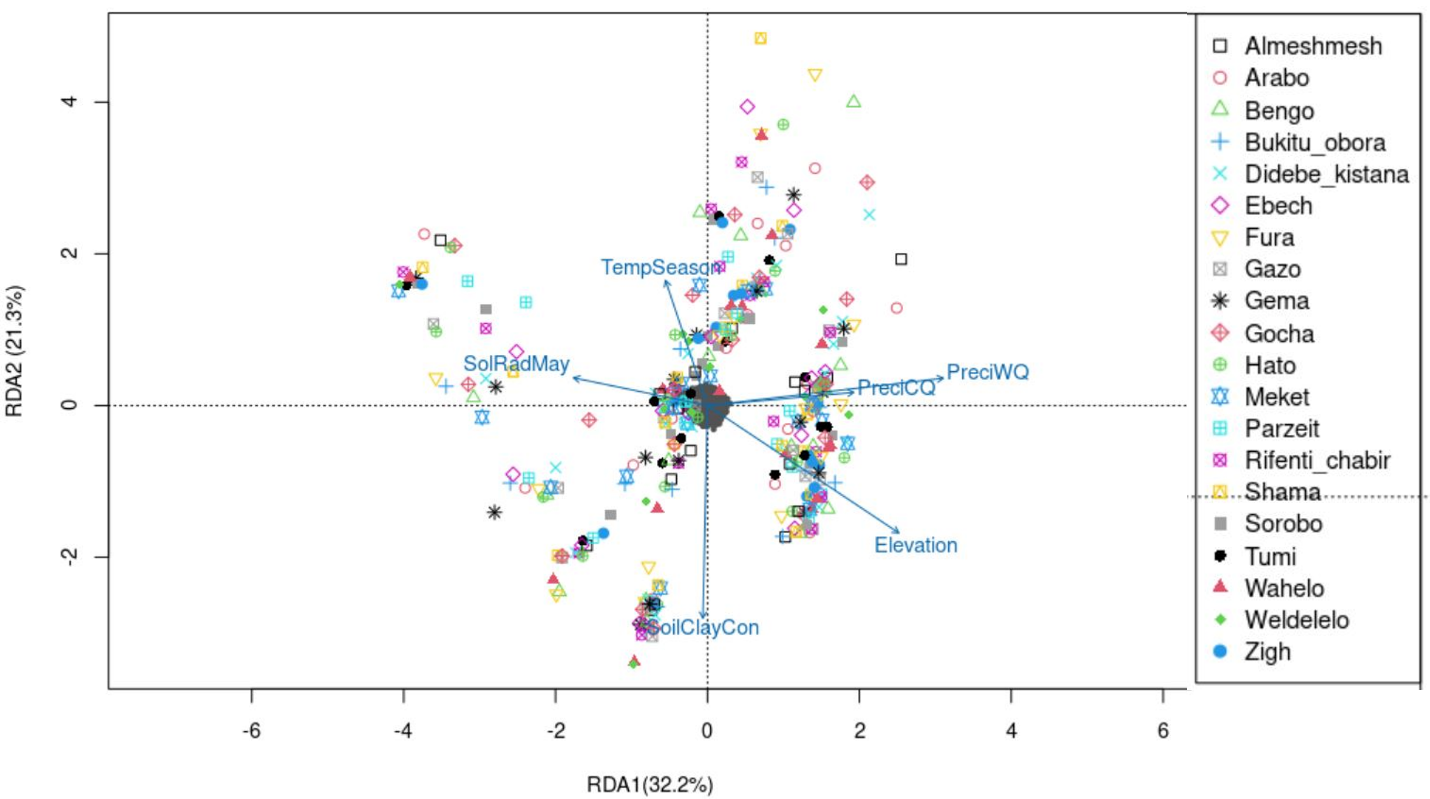


**Supplementary Figure 8.** PCA plot based on RDA axes 1 and 2 of environmental predictors most associated with genotypic variation. PreciWQ=precipitation of the warmest quarter; PrecCQ=precipitation of the coldest quarter; TempSeason=temperature seasonality; SolRadMay=solar radiation of May; SoilClayCon=soil clay content.


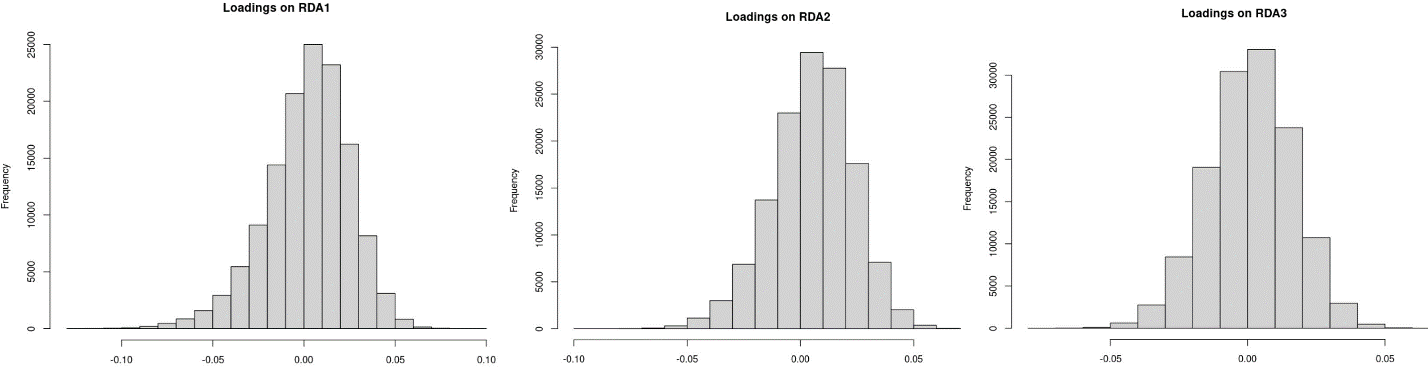


**Supplementary Figure 9.** RDA loadings for environmental predictors


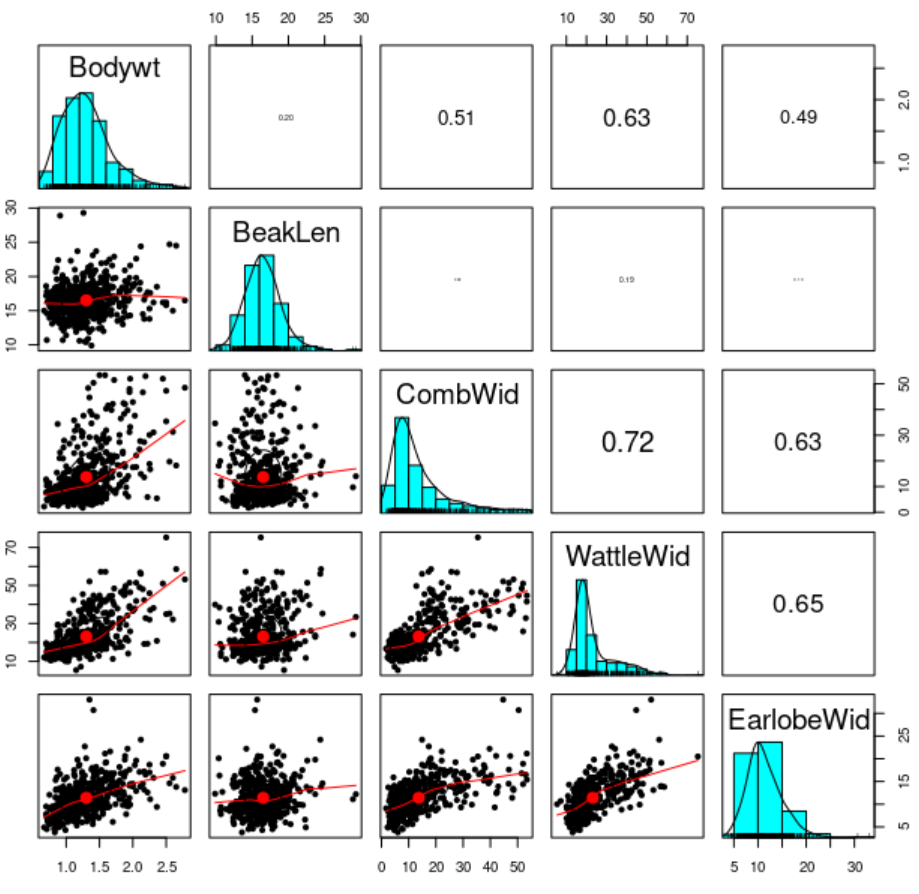


**Supplementary Figure 10.** Correlation among five phenotypic predictors out of eight predictors identified by species and phenotypic distribution models and used for partial RDA.


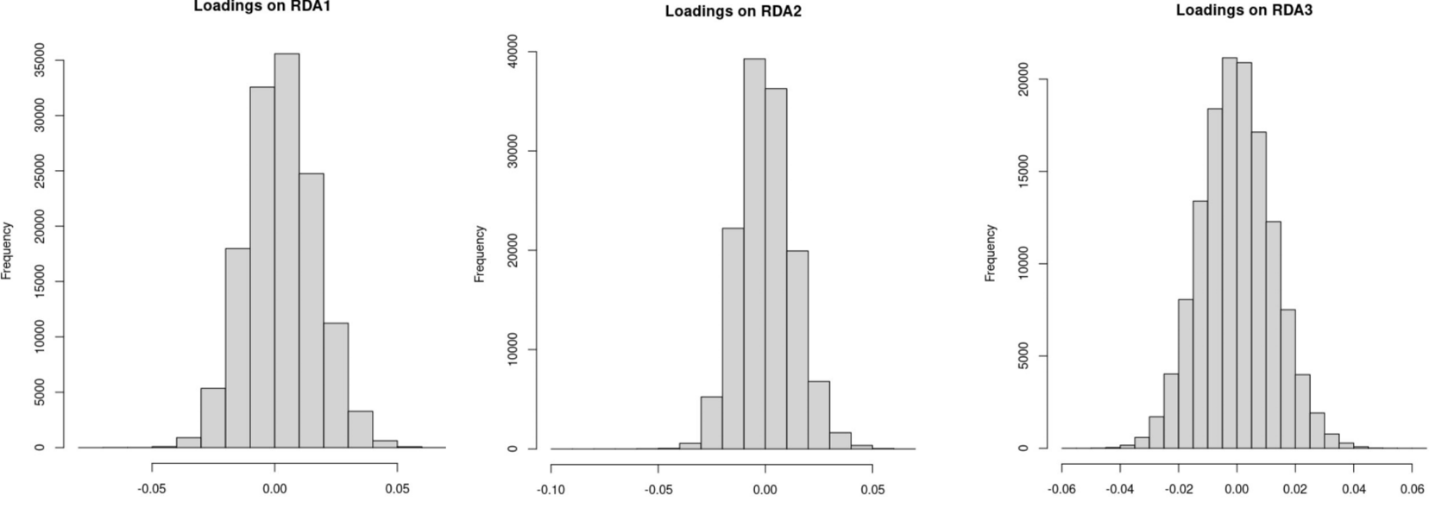


**Supplementary Figure 11.** RDA loadings for phenotypic predictors in gradients *-I, -III*, and *-IV*.


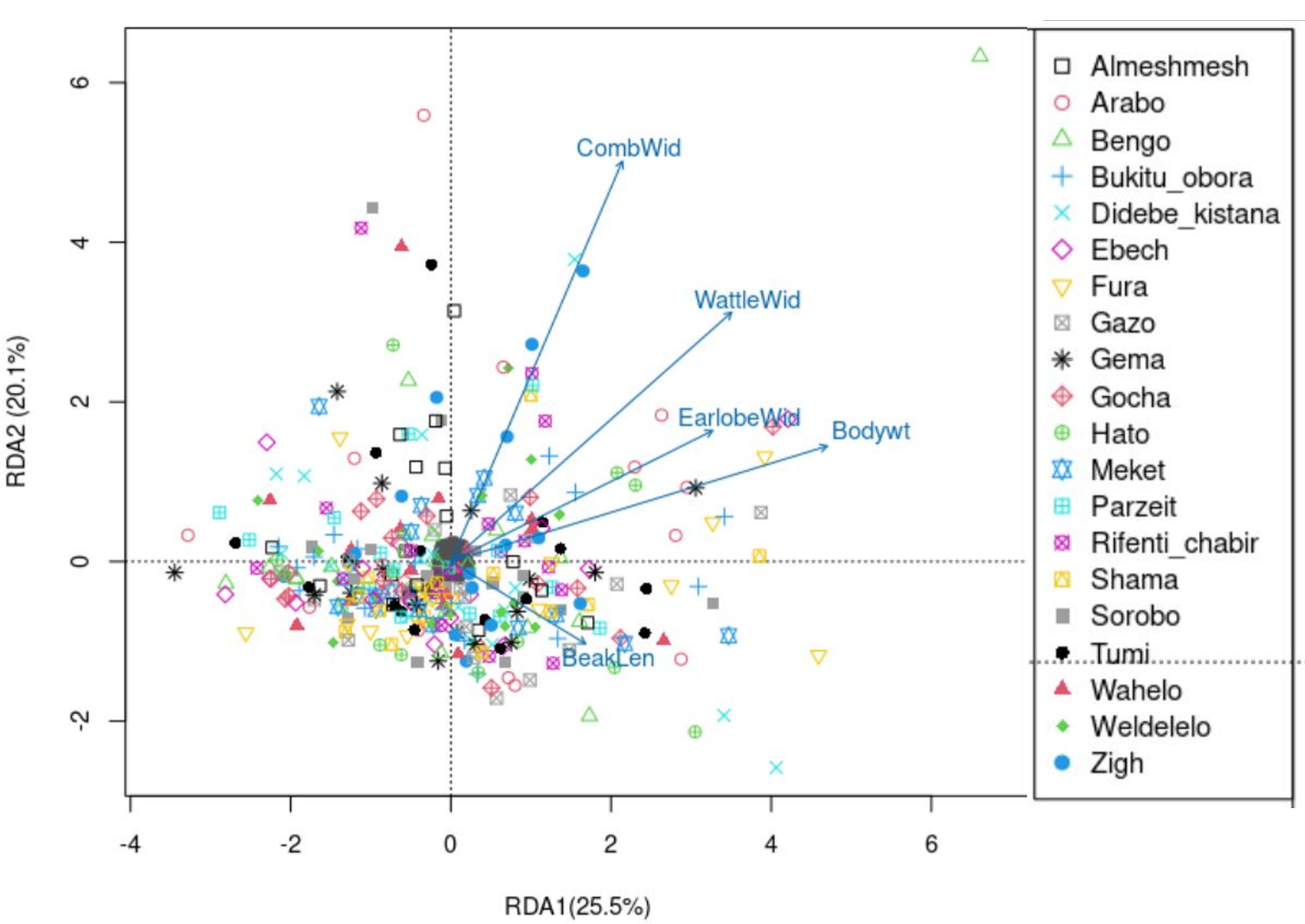


**Supplementary Figure 12.** PCA plot based on RDA axes 1 and 3 for phenotypic predictors


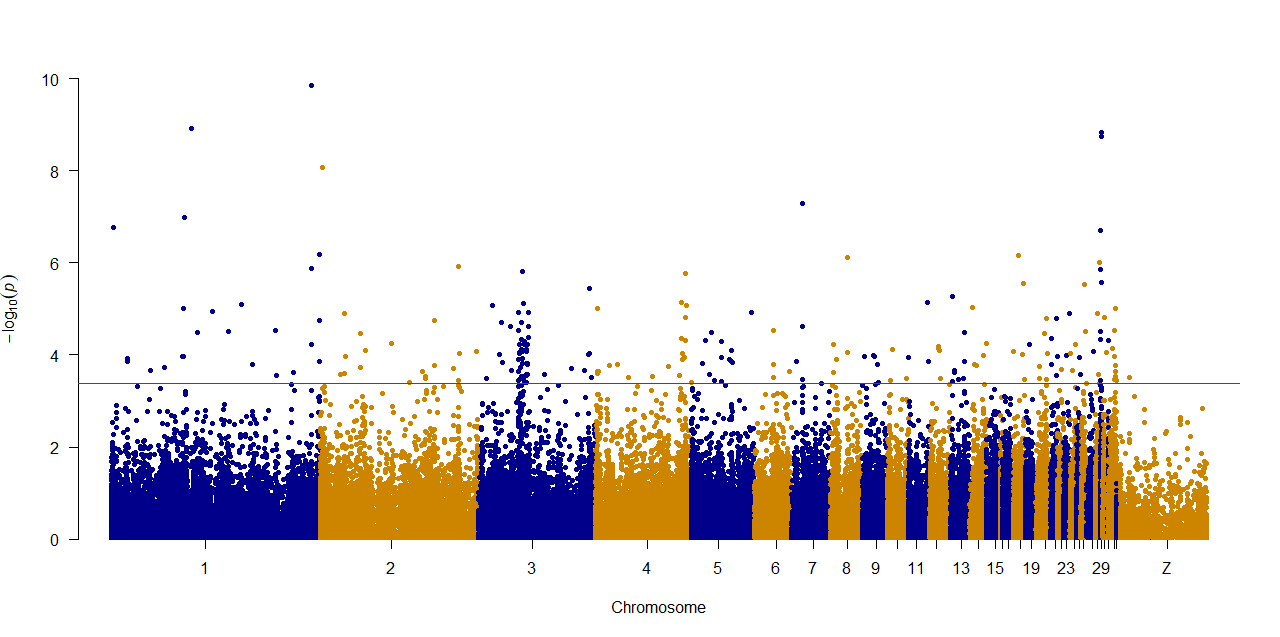
 **Supplementary Figure 13.** Manhattan plot of RDA showing the association of SNPs with phenotypic variation in the five quantitative traits from chickens sampled from populations in *gradient-II*. The y-axis indicates -log 10 (p-value). SNPs with  -log(p-value) higher than 3 are significantly associated (*p* < 0.001) with the traits.


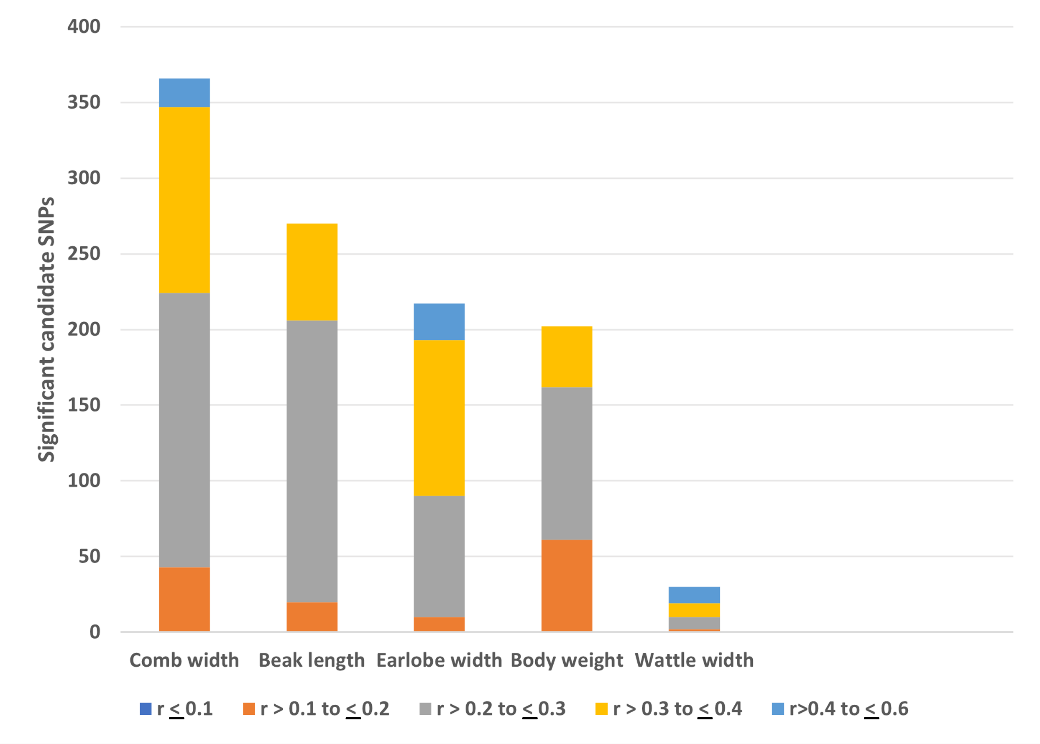


**Supplementary Figure 14** . Stacked bar chart showing the number of outlier SNPs (*p* < 0.01) associated with quantitative traits in *gradients-II* and their splits based on correlation values identified by partial RDA analysis
