# Supplementary material for: Landscape genomics reveals regions associated with adaptive phenotypic and genetic variation in Ethiopian indigenous chickens
Source: BMC Genomics. 2024 Mar 18;25:284. doi: 10.1186/s12864-024-10193-6 (PMC10946127; doi:10.1186/s12864-024-10193-6)
Supplement: Supplementary file 2 — Supplementary Material 2 [file 12864_2024_10193_MOESM2_ESM.docx]

**Supplementary Information**

**Supplementary Tables**

**Supplementary Table 1.** Geographic distribution of 26 Ethiopian indigenous chicken sample populations where phenotypic and genotypic data was collected from

| **Population (code)** | **District** | **Spatial gradient** | **Agroecology** | **Regional State** | **Geolocation** | | **Elevation (m.a.s.l.)** |
| --- | --- | --- | --- | --- | --- | --- | --- |
|  |  |  |  |  | **Long (E)** | **Lat (N)** |  |
| Gazo (1) | Gazo | I | Highland | Amhara | 39.12 | 11.68 | 3175 |
| Meket (2) | Meket | I | Highland | Amhara | 38.75 | 11.72 | 2895 |
| Wahelo (3) | Tehuledere | I | Midaltitude | Amhara | 39.65 | 11.29 | 2150 |
| Weldelelo (4) | Tehuledere | I | Midaltitude | Amhara | 39.71 | 11.26 | 2060 |
| Arabo (7) | Kalu | I | Lowland | Amhara | 39.93 | 11.16 | 1525 |
| Fura (6) | Bati | I | Lowland | Amhara | 40.06 | 11.18 | 1400 |
| Hato (5) | Bati | I | Lowland | Amhara | 40.05 | 11.12 | 1205 |
| Badu (11) | Girawa | II | Highland | Oromia | 41.86 | 9.87 | 2464.5 |
| Birbirsa (10) | Girawa | II | Highland | Oromia | 41.76 | 9.21 | 2437.8 |
| Lafinfedo (12) | Jarso | II | Midaltitude | Oromia | 42.23 | 9.44 | 2175.8 |
| Melkajebdu (13) | Jarso | II | Midaltitude | Oromia | 42.28 | 9.53 | 2017.4 |
| Dalecha (8) | Meiso | II | Lowland | Oromia | 40.65 | 9.21 | 1522.6 |
| Weltane (9) | Meiso | II | Lowland | Oromia | 40.87 | 9.26 | 1295.6 |
| Tumi (15) | Wombera | III | Highland | Benishangul-Gumuz | 35.71 | 10.59 | 2527.8 |
| Ebech (14) | Wombera | III | Midaltitude | Benishangul-Gumuz | 35.71 | 10.64 | 2017.4 |
| Parzeit (17) | Dibate | III | Lowland | Benishangul-Gumuz | 36.23 | 10.67 | 1581.2 |
| Zigh (16) | Dibate | III | Lowland | Benishangul-Gumuz | 36.16 | 10.68 | 1421.4 |
| Almeshmesh (19) | Guba | III | Lowland | Benishangul-Gumuz | 35.3 | 11.8 | 662.7 |
| Bengo (18) | Guba | III | Highland | Benishangul-Gumuz | 35.08 | 11.64 | 407.4 |
| Didibe Kistana (20) | Horro | IV | Highland | Oromia | 37.17 | 9.59 | 2467.7 |
| Burkitu Obora (21) | Horro | IV | Highland | Oromia | 37.04 | 9.5 | 2421 |
| Rifenti Chabir (22) | Horro Buluk | IV | Highland | Oromia | 37.07 | 9.61 | 2710 |
| Gocha (23) | Karat zuria | IV | Lowland | SNNPR | 37.39 | 5.34 | 1583.3 |
| Sorobo (24) | Karat zuria | IV | Lowland | SNNPR | 37.44 | 5.42 | 1241.8 |
| Shama (25) | Chencha | IV | Midaltitude | SNNPR | 37.51 | 6.17 | 2185.7 |
| Gema (26) | Chencha | IV | Highland | SNNPR | 37.65 | 6.29 | 2445.4 |

*****Traditional agroecological classes (AEs) comprise three groups measured in m.a.s.l.: I=lowlands (400-1800); II=1800-2400; III=2400-3500 (Dove, 1890). **^§^** Official AEs represent standard agroecologies of Ethiopia (MoA, 1998).

**Supplementary Table 2.** Selected environmental predictors (climatic, soil, and vegetation type) analysed for the locations of Ethiopian indigenous chicken sample populations

| Type | Variable | Units | Link |
| --- | --- | --- | --- |
| Climatic | BIO3-Isothermality (BIO2/BIO7) (x100) | ^0^C | http://www.worldclim.org/version2 |
|  | BIO4-Temperature seasonality (SD x100) | ^0^C |  |
|  | BIO11-Mean temperature of coldest quarter | ^0^C |  |
|  | BIO18-Precipitation of warmest quarter | mm/m^2^ |  |
|  | BIO19-Precipitation of coldest quarter | mm/m^2^ |  |
|  | Solar radiation month of May | (Kj m^-2^ day^-1^) |  |
|  | Water vapor pressure for the month of May | (kPa) |  |
|  | Water vapor pressure for the month of August | (kPa) |  |
|  | Elevation | m.a.s.l. | http://www.diva-gis.org/datadown |
| Soil | Soil clay content | (weight %) | https://www.isric.org/projects/soil-property-maps-africa-250-m-resolution |

**Supplementary Table 3**. Quantitative traits measured on individual hens (n=113) and cocks (n=113) in indigenous Ethiopian chicken sample populations and identified for their association with habitat suitability and phenotypic differentiation^1^

| **No.** | **Trait** | **Details** | **Unit** | **Equipment** | **Accuracy** |
| --- | --- | --- | --- | --- | --- |
| 1. | Adult live body weight (Bodywt) | fasting weight | g | digital balance | 10g |
| 2. | Beak length (BeakLen) | tip of the beak to insertion of the beak into the skull | mm | ImageJ | 0.1mm |
| 3. | Comb width (CombWid) | from the tip of the central spike until insertion of the comb in the skull | mm | ImageJ | 0.1mm |
| 4. | Wattle width (WattleWid) | from insertion of the right wattle into the beak to the end of the wattle | mm | ImageJ | 0.1mm |
| 5. | Earlobe width (EearlobeWid) | the second maximum dimension | mm | ImageJ | 0.1mm |

^1^Kebede et al (2021)
